# Composition and distribution of fish environmental DNA in an Adirondack watershed

**DOI:** 10.7717/peerj.10539

**Published:** 2021-02-26

**Authors:** Robert S. Cornman, James E. McKenna, Jr., Jennifer A. Fike

**Affiliations:** 1 U.S. Geological Survey, Fort Collins Science Center, Fort Collins, CO, USA; 2 U.S. Geological Survey, Great Lakes Science Center, Cortland, NY, USA

**Keywords:** Environmental DNA, Barcode sequencing, Metagenetics, Computational biology, Mitochondrial 12S ribosomal RNA, New York state, Dam removal, Fisheries restoration

## Abstract

**Background:**

Environmental DNA (eDNA) surveys are appealing options for monitoring aquatic biodiversity. While factors affecting eDNA persistence, capture and amplification have been heavily studied, watershed-scale surveys of fish communities and our confidence in such need further exploration.

**Methods:**

We characterized fish eDNA compositions using rapid, low-volume filtering with replicate and control samples scaled for a single Illumina MiSeq flow cell, using the mitochondrial 12S ribosomal RNA locus for taxonomic profiling. Our goals were to determine: (1) spatiotemporal variation in eDNA abundance, (2) the filtrate needed to achieve strong sequencing libraries, (3) the taxonomic resolution of 12S ribosomal sequences in the study environment, (4) the portion of the expected fish community detectable by 12S sequencing, (5) biases in species recovery, (6) correlations between eDNA compositions and catch per unit effort (CPUE) and (7) the extent that eDNA profiles reflect major watershed features. Our bioinformatic approach included (1) estimation of sequencing error from unambiguous mappings and simulation of taxonomic assignment error under various mapping criteria; (2) binning of species based on inferred assignment error rather than by taxonomic rank; and (3) visualization of mismatch distributions to facilitate discovery of distinct haplotypes attributed to the same reference. Our approach was implemented within the St. Regis River, NY, USA, which supports tribal and recreational fisheries and has been a target of restoration activities. We used a large record of St. Regis-specific observations to validate our assignments.

**Results:**

We found that 300 mL drawn through 25-mm cellulose nitrate filters yielded greater than 5 ng/µL DNA at most sites in summer, which was an approximate threshold for generating strong sequencing libraries in our hands. Using inferred sequence error rates, we binned 12S references for 110 species on a state checklist into 85 single-species bins and seven multispecies bins. Of 48 bins observed by capture survey in the St. Regis, we detected eDNA consistent with 40, with an additional four detections flagged as potential contaminants. Sixteen unobserved species detected by eDNA ranged from plausible to implausible based on distributional data, whereas six observed species had no 12S reference sequence. Summed log-ratio compositions of eDNA-detected taxa correlated with log(CPUE) (Pearson’s *R* = 0.655, *P* < 0.001). Shifts in eDNA composition of several taxa and a genotypic shift in channel catfish (*Ictalurus punctatus*) coincided with the Hogansburg Dam, NY, USA. In summary, a simple filtering apparatus operated by field crews without prior expertise gave useful summaries of eDNA composition with minimal evidence of field contamination. 12S sequencing achieved useful taxonomic resolution despite the short marker length, and data exploration with standard bioinformatic tools clarified taxonomic uncertainty and sources of error.

## Introduction

Compositional surveys of environmental DNA (eDNA) at genetic “barcode” loci are rapidly becoming an alternative or complement to traditional monitoring (Baird & Hajibabaei, 2012). For fisheries management and conservation of aquatic environments, the attractiveness of genetic methods is apparent, as they allow an alternative depiction of communities that are difficult to reconstruct, particularly for cryptic and elusive species. Routine eDNA monitoring could also be more economical and standardized than traditional active-capture approaches. Several studies have shown eDNA detections to be accurate relative to capture methods, in aggregate (Shaw et al., 2016; Hänfling et al., 2016; Evans et al., 2017; Gillet et al., 2018; Pont et al., 2018; Goutte et al., 2020), although agreement is often less on a site-by-site basis (Shaw et al., 2016; Li et al., 2018). On the other hand, eDNA methods have biases and complications of their own. For example, eDNA shedding rates can be highly variable among species, life-history stages, environments, or seasons (Dejean et al., 2011; Pilliod et al., 2014; Olds et al., 2016; Buxton et al., 2017; Klymus et al., 2015; Robson et al., 2016; Kelly et al., 2014; Sassoubre et al., 2016; De Souza et al., 2016; Hayami et al., 2020; Wilcox et al., 2015; Stoeckle, Soboleva & Charlop-Powers 2017). Environmental variables such as water chemistry, temperature, flow, and sediment exchange can strongly influence eDNA capture (Barnes et al., 2014; Jane et al., 2015; Strickler, Fremier & Goldberg, 2015; Barnes & Turner, 2016; Stoeckle et al., 2017; Shogren et al., 2017). eDNA can be transported far from its source population (Pont et al., 2018) and can derive from anthropogenic and natural secondary sources (Merkes et al., 2014; Stoeckle, Soboleva & Charlop-Powers 2017; Cornman et al., 2018; Song, Small & Casman, 2017), complicating interpretation. Amplification biases can strongly skew sequence compositions recovered at ‘barcode’ loci (Kelly, Andrew & Ramón, 2019; Kelly et al., 2014), and multiple loci may be needed to detect most taxa of interest (Shaw et al., 2016; Gillet et al., 2018; Li et al., 2018). How well compositions derived from barcode loci can discriminate ecological signals from environmental and methodological noise therefore remains an area of active investigation, and conclusions may vary across loci, environments, and management objectives (Emilson et al., 2017; Laroche et al., 2017; Berry et al., 2019). Thus, despite the repeated demonstration in principle that eDNA compositions are useful for cataloging fish communities, or potentially providing an index of relative abundance, the practical benefits and limitations of eDNA barcoding must still be judged empirically for specific environments and research objectives on a case-by-case basis.

Here we investigate and validate eDNA barcoding as a monitoring tool in the St. Regis River, NY, USA. A tributary of the St. Lawrence with headwaters in the Adirondack mountains, the St. Regis is approximately 86 miles (138 km) in length and drains approximately 860 mi^2^ (2,200 km^2^). The river supports valuable tribal and recreational fisheries and has been targeted for restoration activities, exemplified by the removal in 2016 of the Hogansburg Dam, NY, USA near the confluence with the St. Lawrence (SRMT Environment Division, 2015) and an Atlantic Salmon reintroduction effort (Great Lakes Restoration Initiative, 2018). Sampling was performed as collateral duty by an existing resource management crew without specialized experience, simply by drawing 300 mL through a filter-tip syringe at each site. Following DNA extraction, 12S ribosomal RNA (“12S” hereafter) amplicon libraries for 72 biological samples, 12 technical replicates and 12 negative control samples were multiplexed on a single MiSeq chip.

From these data, we sought to determine: (1) spatiotemporal variation in eDNA abundance, (2) the filtrate needed to achieve strong sequencing libraries, (3) the taxonomic resolution of 12S ribosomal sequences in the study environment, (4) the portion of the expected fish community detectable by 12S sequencing, (5) biases in taxon recovery, (6) correlations between eDNA compositions and catch per unit effort (CPUE) and (7) the extent that eDNA profiles reflect major watershed features. Our bioinformatic approach included (1) estimation of sequencing error from unambiguous mappings and simulation of taxonomic assignment error under various mapping criteria; (2) binning of species based on inferred assignment error rather than by taxonomic rank; and (3) visualization of mismatch distributions to facilitate discovery of distinct haplotypes attributed to the same reference. We used a large record of St. Regis-specific observations to validate our assignments.

## Methods

### Site selection

**Figure 1:**
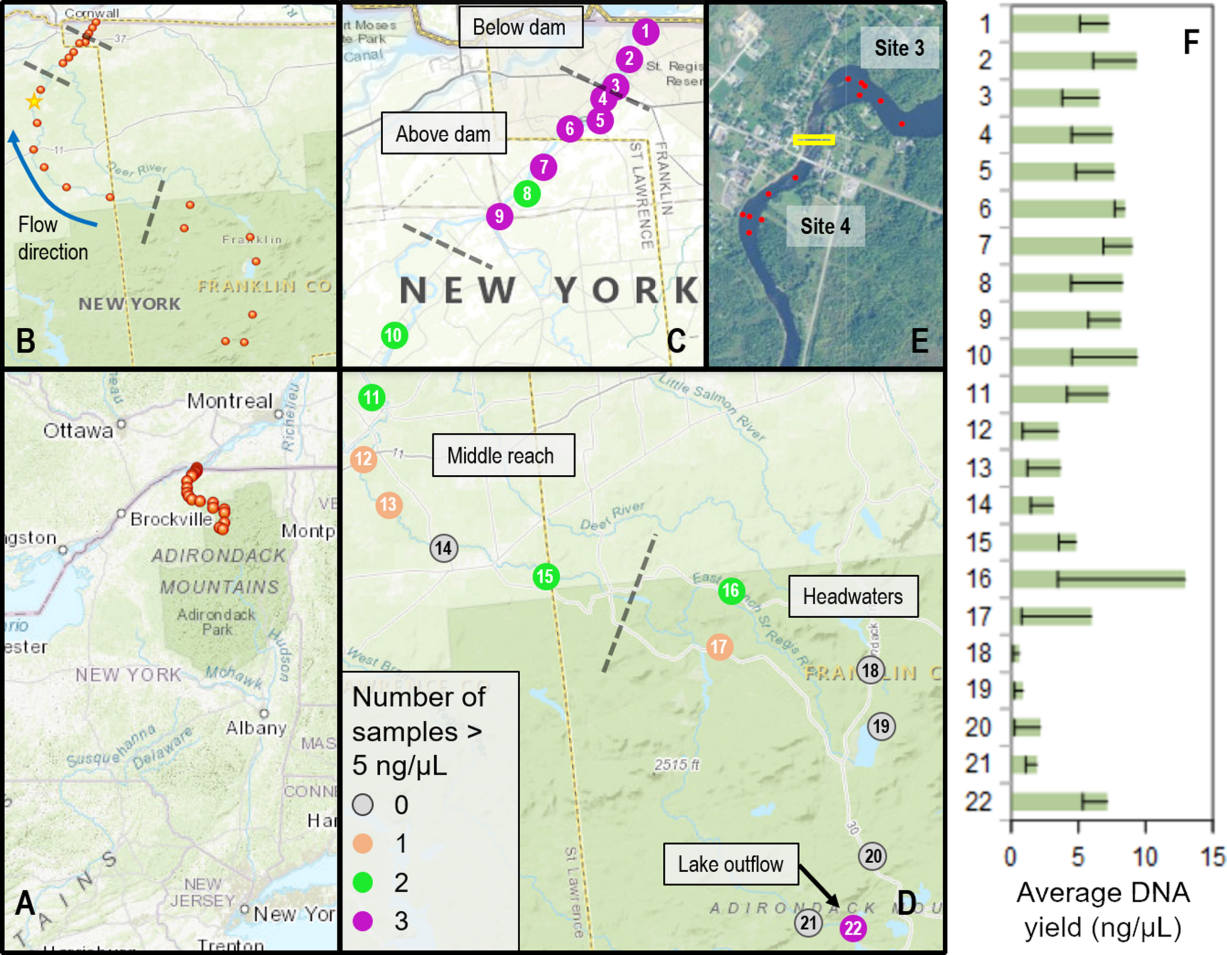
Sampling locations within the St. Regis River watershed and DNA yield. (A) Location of St. Regis sampling sites relative to the northeast United States. (B) Overview showing relative positions of 22 sampling sites from four contiguous regions (separated by dashed lines). The direction of flow northward to the St. Lawrence River is indicated by a blue arrow. The yellow star indicates the location of the USGS water gauge from which flow data was obtained. (C) Numbered site locations in the lower portions of the watershed, above and below the former Hogansburg Dam, NY, USA. (D) Numbered site locations in the remainder of the watershed. The number of samples exceeding 5 ng/uL, an approximate threshold for achieving strong sequencing libraries, is indicated for both (C) and (D) according to the legend. Note the scale of (D) is compressed relative to (C). (E) Detail showing increased sampling at sites 3 and 4 in the vicinity of the Hogansburg Dam, NY, USA (now removed), which is denoted by the yellow box. (F) Average DNA yield at each site, with sample standard error indicated by error bars.

Sites were selected based on access points and ongoing research and management activities, including fish surveys (McKenna et al., 2012; McKenna et al., 2015) (Fig. 1). The river was divided into four contiguous reaches to better capture spatial and ecological variation: (1) three sites from where the St. Regis joined the St. Lawrence River upstream to the (now removed) Hogansburg Dam, NY, USA (“below dam”); (2) six sites from immediately upstream of the Hogansburg Dam, NY, USA to the junction with the Deer River tributary at Helena, NY, USA (“above dam”); (3) six sites from this junction to the main forks of the St. Regis (“middle reach”); and (4) seven sites from diverse headwaters (“headwaters”). Some sites were located on tributaries immediately upstream of the main branch due to access constraints. The final headwaters site was immediately below the outflow of a series of Adirondack Mountain ponds and was expected to represent fish eDNA from these lakes rather than from the St. Regis River, both for comparison and to provide a means of identifying the transport of non-resident eDNA in downstream waters. Within these 22 sites, locations of repeated samples sometimes varied by up to tens of meters, depending on conditions and other activities of the field crew, particularly in the vicinity of the Hogansburg Dam, NY, USA. Additional sampling effort was performed upstream and downstream of this structure (Fig. 1 inset) to evaluate the effect of this barrier on eDNA compositions. Sampling metadata are detailed in Supplemental File S1.

Water samples were obtained at least monthly from each site from August through October, 2015. Sampling events were not conducted during periods of heavy rainfall, and water flow was measured at most sampling events to gauge consistency of flow. Historical flows for the St. Regis River were obtained from the U.S. Geological Survey water gauge at Brasher Center, NY, USA which is upstream of the Hogansburg Dam, NY, USA impoundment (U.S. Geological Survey, 2020b). The location of this gauge is marked on Fig. 1.

### Sample acquisition

Water samples were collected by filtering a total of 300 mL through 25-mm diameter cellulose nitrate filters with a pore size of 0.8 µm (Whatman, Maidstone, UK), using a 50 mL HSW Soft-Ject luer-lock syringe affixed with an unbranded polypropylene luer-lock filter holder. Three subsites were selected to average inputs from near shore, far shore, and mid-channel waters, avoiding turbulent water as well as eddies that might accumulate biofilm. Subsites were accessed by wading or by boat. At each subsite, 100 mL was filtered by twice drawing the maximum 50 mL volume and discharging the filtrate. The intake of the filter holder was placed just under the surface, upstream of all equipment and personnel and after allowing any disturbed sediment to dissipate. The syringe and filter holder were handled with sterile latex gloves and the filter was removed from the filter holder with tweezers that had been stored in 100% ethanol. A freshly sterilized filter holder was used at each site. Field blanks were performed prior to sampling by drawing 300 mL of stock deionized water from a sterile polypropylene bottle. Used filters were placed in a 15 mL centrifuge tube and stored in a cooler on ice until transport to the U.S. Geological Survey, Tunison Aquatic Laboratory, where they were transferred to a freezer rated at −20 °C.

### DNA extraction and barcode sequencing

Filters were transported to the U.S. Geological Survey, Leetown Science Center for DNA extraction using the method of Renshaw et al. (2015). Briefly, whole filters were incubated in a hot hexadecyltrimethylammonium bromide (“CTAB”) buffer, an aliquot of which was then extracted using a phenol-chloroform-isoamyl alcohol procedure. DNA concentration was quantified using a NanoDrop model ND1000 spectrophotometer and then diluted as necessary to a maximum concentration of 2 ng/µL. Extracted DNA was shipped cold to the U.S. Geological Survey, Fort Collins Science Center for library preparation and sequencing.

Amplicons were based on the the 12S-v5 rRNA primers of Riaz et al. (2011). A “preamplification” polymerase chain reaction (PCR) was performed in 25 µL volumes with 2 µL of DNA extract, 0.2 mM of each deoxyribonucleotide triphosphate, 0.5 µM forward primer, 0.5 µM reverse primer, 1.25 U GoTaq Flexi DNA polymerase (Madison, WI, USA), 1.5 mM MgCl_2_, and 1X GoTaq Flexi Buffer (Madison, WI, USA). The thermocycler program used an initial melt step of 95 °C for 2 min, followed by 25 cycles of 95 °C for 45 s, 50 °C for 45 s and 72 °C for 1 min 30 s, followed by a final extension at 72 °C for 2 min. Illumina sequencing adaptors were appended in a second PCR that used 3 µL of the preamplification product as template. Reactions used 0.5 µM of forward and reverse primers, 12.5 µL KAPA2G Fast HotStart ReadyMix (KAPA Biosystems, Wilmington, MA, USA) and 0.25 µL BSA (New England Biolabs, Ipswich, MA, USA) in a 25 µL total volume. The thermocycler program was unchanged from the preamplification. This second PCR was performed in triplicate per sample and subsequently pooled to minimize stochastic amplification noise. Amplicons were evaluated by migrating 5 µL of product in a 2% agarose gel and visualizing product size and intensity relative to a standard with ethidium bromide staining.

Amplification products were then cleaned using the UltraClean HTP 96-well PCR clean-up kit (MoBio, Moscow, Russia) and eluted with 30 µL water. Dual-index Nextera XT barcodes were appended in 50-µL reactions using 5 µL of the cleaned adaptor PCR product, 5 µL of each index, 25 µL of 2xKAPA HiFi HotStart ReadyMix, and 10 µL water. Amplification conditions were 95 °C for 3 min, then 8 cycles of 95 °C for 30 s, 55 °C for 30 s and 72 °C for 30 s, with a final extension at 72 °C for 5 min. The indexed templates were again cleaned using the UltraClean kit and eluted wtih 50 µL water. Samples were quantified using a Qubit High Sensitivity DNA Assay (Life Technologies, Carlsbad, CA, USA) and diluted to a target concentration of 4 nM (samples already below dilution targets, such as negative control samples, were left unaltered). The final library prep was denatured and diluted to a target concentration of 6 pM, spiked with 30% phiX control sequence and sequenced on an Illumina MiSeq with a 600 cycle version 3 chip to produce 300-bp paired reads.

### Read trimming

Paired-end 300-bp reads were produced but only the first read of each pair was analyzed. We did not merge read pairs prior to mapping as a nontrivial fraction may fail to merge correctly and consensus characters are usually not interpretable to downstream applications (e.g., mapping and clustering of reads). Read length, composition, and quality distributions were evaluated before and after processing with FastQC (Andrew, 2020). Read trimming was performed with bbduk of the bbmap package (Bushnell, 2020), specifying a modulus trim of five, minimum length of 50, and a minimum quality of 10 (phred-scaled). Adapter sequences were identified and trimmed using a kmer size of 15 and with the minimum kmer parameter set to 11.

### Reference database generation

The workflow used to assess mapping parameters and assign reads to taxa in the reference database is diagrammed in Fig. S1, which began with the aggregation of a reference database of fish 12S sequences. While capture-based observations of fish assemblages in the St. Regis River have been performed (McKenna et al., 2015), we based our reference taxa on the Cornell checklist of freshwater fishes for the state of New York (http://www2.dnr.cornell.edu/cek7/nyfish/#fishlist, access date 20 January 2020) to make our binning procedure generalizable to regional watersheds. The checklist contained 132 species, ignoring subspecific designations. The taxonomic identifiers associated with these taxa were obtained from the Taxonomy resource of the National Center for Biotechnology Information (NCBI). All DNA sequences less than 50 kb in length associated with these taxonomic identifiers and matching the search text “12S” were downloaded (accessed 22 January 2020). These were aligned with mafft (Katoh et al., 2002), manually reversing the strand and trimming to the primer region as necessary. This set of sequences was then used as a seed to search the nt database to identify additional 12S sequences that may not have been annotated as such (e.g., mitochondrial genomes). Matches to accessions with taxonomies from the checklist were parsed and included in the initial database.

The initial database was then filtered by evaluating phylogenetic consistency, sequence completeness, and sequence redundancy. As the taxonomy assigned to database sequences is submitter supplied and may be incorrect, a neighbor-joining tree of the initial sequences was constructed and evaluated in MegaX (Kumar et al., 2018) using the Kimura two-parameter model. Based on this analysis (Supplemental File S2), we excluded two fathead minnow accessions (AF126360, AF126363) that clustered with other cyprinid minnow species and not conspecific sequences. A 12S sequence extracted from a whole mitochondrial sequence (KC663435) attributed to *Sander canadensis* was also removed as it was >13% divergent from other *S. canadensis* sequences in the NCBI nt database.

As some unique reference sequences were substantially shorter than the full length of the 12S alignment, we investigated whether environmental sequence reads could serve as proxy references. Two partial 12S sequences, one for *Culaea inconstans* (AY283324) and one for *Esox masquinongy* (AY430274), were replaced with exactly and uniquely aligned full-length 12S reads from the data. The reference database was then filtered to exclude sequences with terminal alignment gaps greater than nine positions (excepting lamprey species which have genuinely shorter 12S sequences than Actinopteri). Highly redundant taxa were dereplicated at 100% identity, resulting in 238 references for 110 species.

### Estimation of sequence error rate

Evaluating read-mapping stringency and identifying sets of taxa with high rates of potential misassignment first requires an accurate assessment of per-base sequence error. However, the magnitude and distribution of error along reads can vary from run to run and can also differ for amplicon sequences relative to the phiX spike from which mean error rates are computed by the sequencing software (Coykendall et al., 2019; Iwanowicz et al., 2020). We therefore estimated sequencing error directly from the data using a subset of unambiguously mapped reads. This was accomplished by identifying species that had (1) high genetic distances to other taxa, (2) multiple distinct reference accessions (to better accommodate genuine biological variation), and (3) large numbers of mapped reads (for more robust rate estimation).

We first aligned the reference database to itself with BLASTN and extracted the lowest conspecific and highest heterospecific bit scores for each species. We then mapped all unique reads in the data set (i.e., dereplicated at 100% identity) to the reference database using bowtie2 with “local” and “sensitive-local” parameter switches. We identified three species for which references differed from heterospecific references by an alignment bit score of at least 28 and with high read counts in the data set: largemouth bass (*Micropterus salmoides*), smallmouth bass (*Micropterus dolomieu*) and rock bass (*Ambloplites rupestris*). These species are all members of family Centrarchidae with multiple reference sequences available and that were abundant in regional surveys (McKenna et al., 2015; River Institute, 2019). We tabulated the per-base mismatch distribution for reads mapped to references of these species by parsing the mismatch strings according to the SAM alignment specification (Li et al., 2009). While this estimate potentially conflates natural sequence variation with sequence error, we did not identify any sites with greater than 10% mismatch rate within any reference (excepting *M. salmoides* accession AP014537, which had only four reads mapped and was excluded). We used PAST3 (Hammer, Harper & Ryan, 2001) to fit a polynomial function to reference coordinates 10–90 (Fig. S2), as mismatch rates at the extremes of reads may be idiosyncratic and not generalizable. The best fit polynomial, based on Akaike information criterion score, was order one (linear) with a rate of 0.66 percent at position 1 and a rate of 2.96 percent at position 98.

### Simulation of assignment error based on reference sequences

We used Grinder (Angly et al., 2012) to simulate 10,000 reads for each reference accession with Illumina-like error profiles, specifying a linearly progressive error rate with the values estimated above. Our 5′ value was roughly twice the default value for Illumina reads in the Grinder program, but similar to the 3′ value (Angly et al., 2012). Homopolymer error was ignored as Illumina reads have relatively low homopolymer error and long homopolymers were absent from the 12S references. The simulated reads were then mapped to the reference database with bowtie2, again using the “local” and “sensitive” parameter switches, with only the top match reported. We then tabulated the number of times a simulated read of a given query taxon mapped to a heterospecific accession and represented these taxon pairs with an adjacency matrix. Only query species with total misassignment rates greater than 5% in the simulated data were included, and individual query-reference combinations contributing less than 1% of the total error for that query species were subsequently dropped. Graph connections were then visualized in igraph (Csardi & Tamas, 2006) and taxa within each closed network were binned (Fig. S3). Three bins consisted of cyprinid minnows and four additional bins of related congeners (two *Esox* species, two *Salvelinus* species, three *Alosa* species and three *Acipenser* species). Another four bins were imposed a priori because no sequence differences existed between the available 12S references: three species of the salmonid genera *Coregonus* and *Prosopium*, two pairs of lamprey species, and the suckers *Hypentelium nigricans* and *Moxostsoma anisurum*. These bins resulted in an overall assignment error of 0.83% in the simulated reads, which assumed equal abundance of all species.

### Mapping reads to references and tabulating counts

Mapping reads directly to reference sequences is a popular strategy for assessing taxon abundance because it is computationally fast and the references are vouchered rather than inferred haplotypes. On the other hand, the small edit distances between species at the 12S locus and the potential for reference sequence error and novel variation in environmental sequences make the choice of mapping stringency difficult. We therefore employed an unconventional read counting approach specifically to evaluate the sensitivity of counts to various alignment characteristics. Our approach is based on common alignment-based software and scoring conventions as clustering programs typically do not report these variables. While we do not argue our approach is either optimal or efficient, it permits an exploration of the factors affecting perceived read abundances so that reference databases can be improved.

We began by local mapping reads to references as this approach tolerates artifacts that can occur at read edges, due to incomplete adapter trimming or truncated reference sequences, for example (Iwanowicz et al., 2016), while still reporting the length and location of the skipped portions. We then tabulated three alignment characteristics for each mapped read to evaluate as thresholds for mapping stringency: the number of distinct gap positions (“*G*”), the number of mismatch positions (“*M*”) and the difference between the number of aligned bases and the reference length (“*L*”). Skips at read edges were counted as a single gap position. The *G* and *L* values are distinct in that *L* prevents alignments that have long skips or between references and truncated reads. The *G*, *L* and *M* values for each alignment were parsed from the bowtie2 output according to the SAM sequence alignment specification (Li et al., 2009), using the code in Supplemental File S3.

The number of correctly assigned reads summed over all species is shown in Fig. S4 for various threshold combinations. Based on overall error rates for the simulated reads, we initially chose to set the mapping stringency thresholds to *G* = 1, *M* = 3 and *L* = 3. For the *G* and *M* thresholds, there was negligible increase in the number of reads mapped at higher values but substantial gain over lower values. However, there was no comparable flattening of the curves across the range of *L* values considered (2–5). We therefore selected *L* = 3 because more permissive gave diminishing returns of sensitivity versus specificity. However, when we applied these values to filter alignments from environmental samples, several taxa that were largely absent at *G* = 1, *M* = 3, *L* = 3 were detected at high levels when the gap threshold G was increased to two or three (Fig. S5). This effect appears to be due to indels in some reference sequences relative to the recovered sequence reads, which would not be apparent in data simulated from those references. We therefore chose *G* = 3, *M* = 3 and *L* = 3 as the mapping stringency parameters to better accommodate indel variation (indel errors can occur in Sanger sequenced reference amplicons due to stutter peaks, for example).

### Observation records for validation

Catch data were aggregated from previous active capture surveys in the St. Regis (McKenna et al., 2012; McKenna et al., 2015) that emphasized habitats from the confluence with the St. Lawrence River to Brasher Falls, NY, USA. Surveys used both model-guided and random procedures to improve the completeness and objectivity of species assessments, following methods of McKenna et al. (2012) and McKenna et al. (2015). Shallow habitats with slow-flowing waters and relatively smooth bottoms were sampled with two 15 m sweeps of a seine (9.1 m in length with 6.35 mm mesh wings and 3.175 mm mesh bag (McKenna et al., 2013)). Headwaters and sites with higher flow or more rugged bottoms were sampled using a single-pass, backpack-electrofishing technique within a 50 m reach, adjusted for sampling efficiency using the methods of McKenna et al. (2015). Deep waters were sampled with boat electrofishing (approximately 100 m transects with electrode settings at 120 or 60 pulses/second at up to 500 volts with output current from 6 to 9 amps) or gillnets (25.4–76.8 mm paneled monofilament mesh, 38 m in length, 1.8 m in height). Collected fish were identified, counted, and released alive, unless needed for voucher. Questionable fish identifications were verified by taxonomists at the New York State Museum and identification errors corrected as necessary. Species abundances for each gear type (seine, gillnet and electroshock) were calculated separately and abundances were standardized to CPUE (number per 100 m^2^ of sample area). Fish observation records for the St. Regis from the New York Department of Environmental Conservation database that used similar collection gear was standardized to CPUE and included in the validation data set (New York Department of Environmental Conservation, 2011).

### Evaluation of multiple haplotypes within mapped reads

Direct mapping to references may conflate multiple unique haplotypes that are within the thresholds chosen (as may happen with de novo clustering as well), particularly when the reference database is incomplete. To evaluate this occurrence retrospectively, we extracted alignment files of all unique reads with the reference to which they were mapped and plotted a histogram of mismatch counts within those alignments. The expected distribution is unimodal when haplotype variation is absent and mismatches are due only to sequence error. When haplotype variation is nontrivial, the observed distribution of mismatches will typically be multimodal unless haplotypes are equidistant from the reference. Multimodal alignments were manually inspected with the Tablet alignment viewer (Milne et al., 2010) to identify reads representative of novel haplotypes.

### Contamination analysis and data censoring

The number of reads mapped to each reference in each sample was summed by taxonomic bin to generate a raw counts table. Because sample crosstalk occurs in Illumina MiSeq sequencing, even when dual indexes are used (Olds et al., 2016), it is advisable to censor cells with very low counts to avoid inflating taxon prevalence and sample diversity. We therefore compared the distribution of taxon abundances in the negative controls with that in the data set as a whole to estimate a background rate of demultiplex error (Fig. S6). Most taxa were either absent from negative controls or present at a rate that scaled log-linearly with total counts, asymptotically approaching an occurrence rate of approximately 0.01%, similar to what has been reported elsewhere (Olds et al., 2016). However, we identified four taxa that were enriched in the negative controls in excess of what can be explain by stochastic demultiplex error. For these taxa, excess reads were generally associated with two of the negative control samples. This pattern indicates point occurrence rather than systematic contamination. The strongest instances of contamination were of brook trout (*Salvelinus fortinalis*) and Atlantic salmon (*Salmo salar*), species routinely handled by both the Tunison Aquatic Laboratory (where samples were stored) and the Leetown Science Center (where DNA was extracted). As these contamination events impacted single control samples, it is impossible to determine which biological samples, if any, were also contaminated. The field crew had noted that one of the 12 negative controls had potentially been contaminated by neglecting to sterilize the tweezers prior to handling the filter. This negative control sample is marked with a star in Fig. S6 and is in fact one of two negative controls that appeared to have been contaminated, either in the field as suspected or during subsequent processing. Atlantic salmon are not known to be established in the St. Regis River at the time of sampling but are at least transiently present due to reintroduction efforts (Great Lakes Restoration Initiative, 2018); reads were detected in only a few biological samples at low rates (see “Results”). In contrast, brook trout occurred in biological samples at moderate abundance and at sites consistent with their habitat requirements. Nonetheless, for the purpose of assessing taxon and sample correlations, sample richness, and other comparative analyses, we removed all four contaminant taxa.

### Taxon validation and database revision

We evaluated the consistency and accuracy of initial eDNA assignments in multiple ways. We first evaluated the pairwise correlation matrix of transformed compositions to identify potentially conflated taxonomic bins, restricting this analysis to taxa detected in at least four samples at a proportion of at least 0.1% (Fig. S7). The strongest positive correlation was found between the congeners *Percina caprodes* and *Percina macrocephala*, suggesting that the two were conflated in the counts table even though their respective reference sequences differ by two substitutions. Inspection of the read pileup confirmed that reads mapping to the two references represented a single consensus haplotype that differed by one substitution and one indel from each of the two references, thus mapping to either with approximately equal likelihood (Fig. S8). The two species were therefore combined into a single bin, although *P. macrocephala* (longhead darter) is a rare endemic of the Ohio River basin that does not occur in the St. Regis (Pennsylvania Natural Heritage Program, 2020) whereas *P. caprodes* (common logperch) is widespread in the eastern U.S. (U.S. Geological Survey, 2020a) and is recorded from the St. Regis River (Table 1). We conclude that only *P. caprodes* was detected but additional voucher sequencing is needed for these *Percina* species to confirm this.

**Table 1:** Taxonomic bins identified by both eDNA and active capture surveys. Total counts are equal to the sum of reads mapped to all accessions of the corresponding taxon, without normalization by sample library size. Average composition is calculated across positive samples only. A sample was considered positive for the taxon if the proportion of reads attributable to the taxon was 0.1% of the total for that library. CPUE is counts per unit effort (numbers per 100 m^2^ of sample area) and are summed across component species of each multispecies bin.

eDNA taxon	Total counts	Summed compositions	Average composition	Number of positive samples*	eDNA rank	Observed taxa	CPUE**	CPUE rank
*Moxostoma anisurum*	809,373	106.367	2.474	43	1	*Moxostoma anisurum*	2.555	9
*Semotilus atromaculatus*	292,352	95.445	2.220	43	2	*Semotilus atromaculatus*	4.134	7
CYPRINID2	286,456	79.818	1.995	40	3	*Luxilus cornutus*, *Notropis heterodon*, *Notropis heterolepis*, *Pimephales notatus*, *Notropis volucellus*, *Notropis atherinoides*	66.947	1
*Micropterus dolomieu*	211,714	73.247	1.878	39	4	*Micropterus dolomieu*	9.751	2
*Ambloplites rupestris*	186,779	69.235	1.871	37	5	*Ambloplites rupestris*	6.225	4
*Ictalurus punctatus*	182,979	59.982	1.935	31	6	*Ictalurus punctatus*	0.964	21
*Etheostoma flabellare*	127,742	54.875	1.892	29	7	*Etheostoma flabellare*	0.153	36
*Percina caprodes*	94,546	48.174	1.784	27	8	*Percina caprodes*	1.977	11
*Cyprinella spiloptera*	101,071	40.770	1.853	22	9	*Cyprinella spiloptera*	6.906	3
*Sander vitreus*	104,568	32.707	1.817	18	10	*Sander vitreus*	1.254	17
*Lepomis gibbosus*	67,622	30.705	1.335	23	11	*Lepomis gibbosus*	1.938	13
*Etheostoma nigrum*	36,510	29.237	1.271	23	12	*Etheostoma nigrum*	0.835	22
CYPRINID3	13,704	27.870	0.845	33	13	*Notropis hudsonius*, *Hybognathus regius*, *Notropis bifrenatus*	0.820	24
*Rhinichthys cataractae*	40,552	23.362	1.947	12	14	*Rhinichthys cataractae*	0.565	26
*Ameiurus nebulosus*	15,700	16.997	1.416	12	15	*Ameiurus nebulosus*	0.625	25
*Catostomus commersonii*	22,848	15.034	1.670	9	16	*Catostomus commersonii*	1.454	16
*Esox lucius*	15,440	14.722	1.338	11	17	*Esox lucius*	0.140	38
*Culaea inconstans*	19,210	14.372	1.796	8	18	*Culaea inconstans*	0.517	27
ACIPENSER	19,681	13.435	1.493	9	19	*Acipenser fulvescens*	1.710	14
CYPRINID1	9,923	12.651	2.108	6	20	Chrosomus neogaeus, *Chrosomus eos*	1.152	18
*Etheostoma olmstedi*	24,478	11.911	1.702	7	21	*Etheostoma olmstedi*	1.965	12
*Notemigonus crysoleucas*	7,242	9.109	1.822	5	22	*Notemigonus crysoleucas*	0.981	20
*Micropterus salmoides*	14,773	8.776	1.755	5	23	*Micropterus salmoides*	0.129	39
*Exoglossum maxillingua*	4,698	7.805	1.951	4	24	*Exoglossum maxillingua*	0.194	35
*Cyprinus carpio*	23,540	7.494	1.874	4	25	*Cyprinus carpio*	0.071	42
*Perca flavescens*	14,713	7.031	1.758	4	26	*Perca flavescens*	3.636	8
*Esox masquinongy*	8,038	6.337	1.267	5	27	*Esox masquinongy*	0.277	30
ICHTHYOMYZON	2,082	3.673	1.224	3	28	*Ichthyomyzon fossor*, Ichthyomyzon spp.	NA	NA
*Pimephales promelas*	72	3.118	1.559	2	29	*Pimephales promelas*	0.265	32
*Pomoxis nigromaculatus*	1,996	1.605	1.605	1	30	*Pomoxis nigromaculatus*	0.061	43
*Noturus gyrinus*	1,840	1.519	1.519	1	31	*Noturus gyrinus*	NA	NA
*Ammocrypta pellucida*	454	1.282	1.282	1	32	*Ammocrypta pellucida*	0.246	33
*Umbra limi*	574	1.161	1.161	1	33	*Umbra limi*	0.142	37
*Etheostoma exile*	1	below threshold	0.000	0	not ranked	*Etheostoma exile*	0.018	46
LETHENTERON-PETROMYZON	9	below threshold	0.000	0	not ranked	Lethenteron spp., *Petromyzon marinus*	0.001	50
*Morone americana*	1	below threshold	0.000	0	not ranked	*Morone americana*	0.006	47
*Notropis stramineus*	4	below threshold	0.000	0	not ranked	*Notropis stramineus*	2.299	10
*Noturus flavus*	1	below threshold	0.000	0	not ranked	*Noturus flavus*	NA	NA
*Percina copelandi*	83	below threshold	0.000	0	not ranked	*Percina copelandi*	0.128	40
*Salmo trutta*	20	below threshold	0.000	0	not ranked	*Salmo trutta*	0.006	48

**Notes:**

* Presence above a 0.1% threshold proportion of counts, out of 45 samples with at least 1,500 counts.

** CPUE, catch per unit effort. For multispecies bins, CPUE was summed across species within the bin.

Another case of potentially conflated taxa involved common shiner (*Notropis volucellus*), emerald shiner (*Notropis atherinoides*), and members of the CYPRINID2 bin (Fig. S7). *N. atherinoides* was a common eDNA assignment but not observed in capture surveys and compositions were strongly correlated with the CYPRINID2 bin. In contrast, *N. volucellus* was the most commonly detected species by CPUE but was weakly detected by eDNA (Table 1). Inspection of read mappings with Tablet and re-clustering of mapped reads with vsearch revealed a single high abundance haplotype that was a single edit distance from references of five species (Fig. S9), three of which had been binned within CYPRINID2 (*Luxilus cornutus*, *Notropis heterodon* and *Notropis heterolepis*). We therefore revised this bin to also include *N. atherinoides* and *N. volucellus* but conclude that if the single, abundant haplotype derives from a single species, it is most likely *N. volucellus*.

In addition to reviewing highly correlated pairs, we also revised taxonomic bins based on apparent haplotype variation within the pool of reads mapped to a reference. The number of mismatches in dereplicated reads aligned to each reference accession was typically unimodal in the allowable range of 0–3 (Fig. S10), as expected if no underlying haplotype structure exists in the pool of mapped reads. However, several multimodal mismatch patterns were evident (marked in Fig. S10) that revealed underlying haplotype variation when investigated post hoc. These patterns of divergence were associated with references for channel catfish (*Ictalurus punctatus*), tessellated darter (*Etheostoma olmstedi*), pumpkinseed (*Lepomis gibbosus*) and brook stickleback (*Culaea inconstans*). BLASTN searches of sequences selected manually with Tablet did not indicate an alternative taxonomy for the haplotypes aligned to *I. punctatus* or *C. inconstans* references (Fig. S11), suggesting they are intraspecific variants. In contrast, the alternative haplotypes identified in reads mapping to *E. olmstedi* did appear to include a congener not listed on the state-wide checklist: one alternative haplotype matched an accession of johnny darter (*E. nigrum*), which has been observed in the St. Regis (Table 1). A second *Etheostoma* haplotype could not be definitively identified and is designated “Etheostoma haplotype 1” hereafter (Fig. S11). Two distinct *Lepomis* haplotypes were identified (referred to hereafter as “*Lepomis* haplotype 1” and “*Lepomis* haplotype 2”) that occurred at low abundance but had a different sample distribution than *L. gibbosus*. The two alternative haplotypes shared two substitutions relative to reference *L. gibbosus* sequences, with an additional two substitutions in the (rarer) *Lepomis* haplotype 2 (note this haplotype could map to a *L. gibbosus* reference despite differing by four substitutions because the first substitution position was within the allowed edge skip).

We also evaluated eDNA detections by comparison to extensive observations derived by active capture (i.e., electrofishing, gillnetting, and seining). Taxa identified by eDNA that were consistent with direct observations are shown in Table 1. Of the 40 concordant bins, 34 were detected by more than 50 sequence reads in total but three were supported by single reads only. Taxa identified by one approach but not definitively by the other are summarized in Table 2. It is unclear whether Atlantic salmon (*Salmo salar*) is currently extant in the watershed, but we have included it as observed in Table 2 based on recent reintroduction efforts. *S. salar* is one of four taxa that were ambiguously detected by eDNA because they were also enriched in the negative control samples (Fig. S6). Six additional species observed by capture methods could not be evaluated by 12S eDNA sequencing as no reference sequences were available. Most of the 16 species detected by eDNA but not observed in the St. Regis capture data were at very low abundance: seven had fewer than ten total reads, with three more having fewer than 50 reads. We manually grouped these uncorroborated detections into three categories of plausibility based on their known ranges and historical patterns (Table 2). The *L. auritus*-matching haplotype (Fig. S11) exceeded the minimum count threshold only at site 22, which was located at the immediate outflow of a series of ponds in the extreme headwaters of the watershed. *L. auritus* may therefore be restricted to these ponds and not occur in the river itself, although infrequent collections of *L. auritus* from the St. Regis watershed have been reported (Fuller, 2021), despite being nominally outside the species range. Note some caught fish were not identified to species and were classified at genus or family level, but these higher-rank assignments were all consistent with eDNA results.

**Table 2:** Discordance between eDNA detections and active capture surveys. Total counts are equal to the sum of reads mapped to all accessions of the corresponding taxon, without normalization by sample library size (applicable for potential contaminants only). CPUE is catch per unit effort (numbers per 100 m^2^ of sample area) and are summed across component species of each multispecies bin. CPUE rank is based on all taxa observed by active capture, not only those discordant with eDNA.

eDNA bin	Total counts	Catch taxa	CPUE*	CPUE rank	Comments
*Rhinichthys atratulus*	1,104	*Rhinichthys atratulus*	1.665	15	eDNA enriched in negative controls
SALVELINUS	10,972	*Salvelinus fontinalis*	0.832	23	eDNA enriched in negative controls
*Cottus cognatus*	66	*Cottus cognatus*	0.021	44	eDNA enriched in negative controls
*Salmo salar*	745	*Salmo salar*	0.019	45	eDNA enriched in negative controls
*Anguilla rostrata*	Not found	*Anguilla rostrata*	0.001	51	
*Fundulus diaphanus*	Not found	*Fundulus diaphanus*	0.078	41	
*Lepisosteus osseus*	Not found	*Lepisosteus osseus*	1.110	19	
*Lepomis macrochirus*	Not found	*Lepomis macrochirus*	0.300	29	
*Hybognathus hankinsoni*	No reference	*Hybognathus hankinsoni*	0.494	28	
*Labidesthes sicculus*	No reference	*Labidesthes sicculus*	0.270	31	
*Moxostoma macrolepidotum*	No reference	*Moxostoma macrolepidotum*	0.234	34	
*Moxostoma valenciennesi*	No reference	*Moxostoma valenciennesi*	0.006	49	
*Notropis rubellus*	No reference	*Notropis rubellus*	4.875	6	
*Semotilus corporalis*	No reference	*Semotilus corporalis*	5.023	5	
*Ameiurus melas*	735	*Ameiurus melas*	Not found		Consistent with range data
*Catostomus catostomus*	227	*Catostomus catostomus*	Not found		Consistent with range data
*Lepomis auritus*	416	*Lepomis auritus*	Not found		Consistent with range data
*Oncorhynchus mykiss*	31	*Oncorhynchus mykiss*	Not found		Consistent with range data
*Campostoma anomalum*	3	*Campostoma anomalum*	Not found		Possible but unlikely
*Carassius auratus*	1	*Carassius auratus*	Not found		Possible but unlikely
*Clinostomus elongatus*	4	*Clinostomus elongatus*	Not found		Possible but unlikely
*Noturus insignis*	1	*Noturus insignis*	Not found		Possible but unlikely
*Oncorhynchus tshawytscha*	1,642	*Oncorhynchus tshawytscha*	Not found		Possible but unlikely
*Sander canadensis*	24	*Sander canadensis*	Not found		Possible but unlikely
*Ameiurus catus*	341	*Ameiurus catus*	Not found		Contradicts range data
*Etheostoma caeruleum*	4	*Etheostoma caeruleum*	Not found		Contradicts range data
*Exoglossum laurae*	6	*Exoglossum laurae*	Not found		Contradicts range data
*Hybopsis amblops*	15	*Hybopsis amblops*	Not found		Contradicts range data
*Nocomis biguttatus*	2	*Nocomis biguttatus*	Not found		Contradicts range data
*Percina maculata*	395	*Percina maculata*	Not found		Contradicts range data

**Note:**

* CPUE, catch per unit effort (number/100 m^2^).

Four taxa were observed by active capture that were not detected at all by eDNA sequencing: banded killifish (*Fundulus diaphanus*), American eel (*Anguilla rostrata*), bluegill (*Lepomis macrochirus*) and long-nosed gar (*Lepisosteus osseus*). To evaluate whether primer-site divergence contributed to this deficit, we downloaded complete mitochondrial genomes for the missing species but identified no primer mismatches (Fig. S12). We conclude that primer mismatch is not a general explanation for the non-detection of these taxa.

The final reference database (Supplemental File S4) contained 244 sequences with the additional reference sequences for *E. nigrum* and the novel haplotypes of *I. punctatus*, *C. inconstans*, *Etheostoma* and *Lepomis*. With the taxonomic binning revised as described above, we repeated the mapping and counting procedure to produce a final counts table (Supplemental File S5).

### Residual read analysis

Reads that failed to align to a reference at the chosen stringency were clustered into operational taxonomic units (OTUs) with vsearch at 98% identity using definition “1” of that software (Rognes et al., 2016). Singleton clusters were discarded and the remainder searched against the nt database and classified using the lowest common ancestor (LCA) method (Huson et al., 2007) based on the top 3% of matches by bit score. For species-level assignment, the best bit score of the sequence was required to be at least 165 and the average percent identity of retained matches to be 97%. For genus-level assignments, these values were 150 and 95%, respectively. The purpose of this analysis was to identify the likely sources of reads that did not map stringently to the reference database, such as taxa missing from the reference database or off-target taxa. The abundances of these OTUs were not tabulated at the sample level and were only used as a quality-control measure for the data set as a whole. A large majority (92.5%) of these unmapped reads that could be assigned a taxonomy by LCA were assigned to class Actinopteri, with relatively few non-fish amplicons detected (Fig. S13). No species present on the state checklist was identified by LCA that was not also identified by direct mapping (Supplemental File S6).

### Data analysis

Diversity was analyzed with the R package vegan (Paradis, Claude & Strimmer, 2004) using raw counts whereas linear statistical analyses of compositions were performed after centered log-ratio transformation of sample proportions and addition of a scalar to render all detections positive in sign. PAST3 (Hammer, Harper & Ryan, 2001) was used for violin plots and ordination. Correlation matrices were plotted with the corrplot package (Wei & Simko, 2017). Any use of trade, firm, or product names is for descriptive purposes only and does not imply endorsement by the U.S. Government.

## Results

### DNA yield and sequencing output

**Figure 2:**
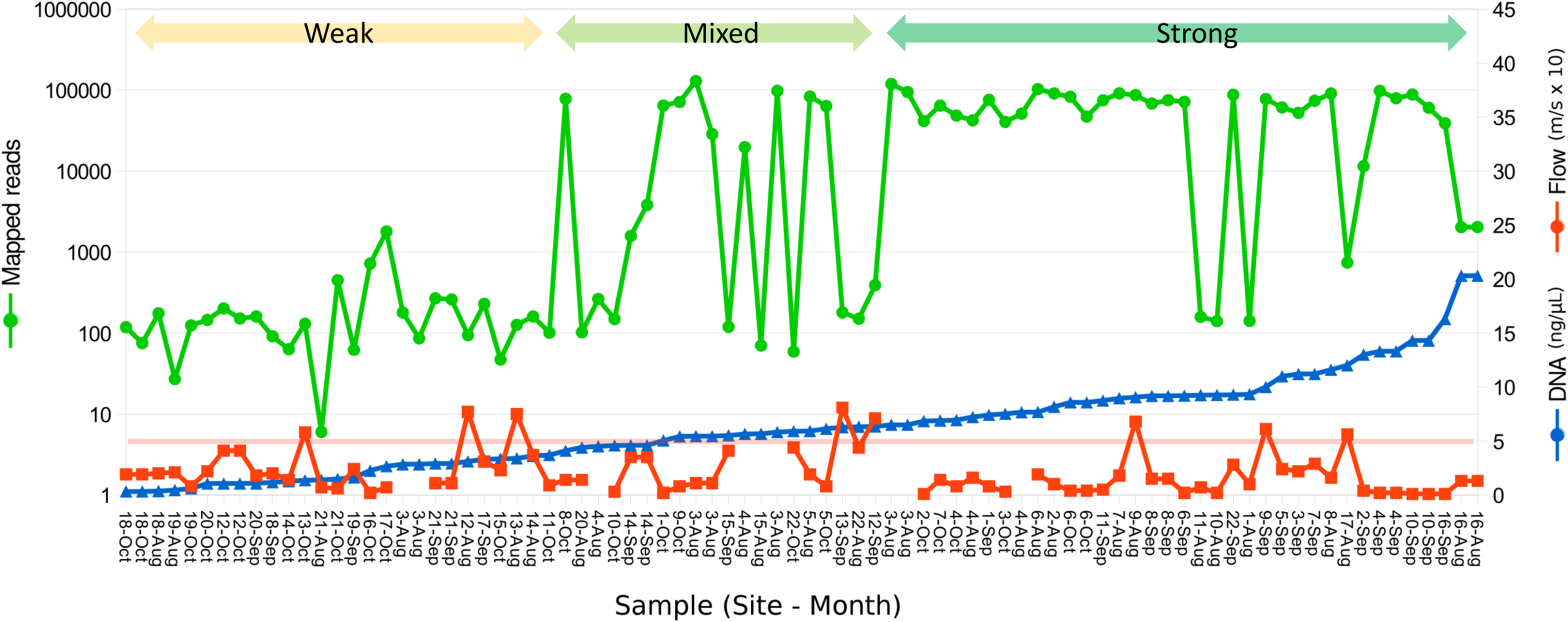
Relation between eDNA concentration and library yield. Library yield is shown on the primary axis and eDNA concentration and flow are shown on the secondary axis, with samples sorted by increasing library yield. Samples are grouped subjectively into three categories of library strength. The red horizontal line corresponds to an initial total DNA concentration of 5 ng/µL, as a reference. Samples greater than 2 ng/µL were diluted to that value prior to the 12S preamplification reaction.

We collected a total of 72 biological samples (Supplemental File S1) from 22 sites (Fig. 1) from August to October, 2015, as well as 12 field blanks (negative controls). Historical data confirmed that this period typically has lower flows and lower daily variance than other months (Fig. S14). DNA concentrations ranged from 0.324 to 20.3 ng/µL per eluted extract and generally increased from headwater sites to middle reach sites to lower reach sites (Fig. 1). There was no apparent relationship between measured flow and DNA yield (Fig. 2), although middle reach and headwater sites showed more within- and among-site variation in DNA recovery (Fig. 1). DNA yield was significantly lower in October (Fig. S14) than in August and September (*P* = 0.002 by ANOVA, with Tukey’s pairwise comparisons significant at *P* = 0.040 and *P* = 0.0018, respectively; see Supplemental File S7 for full test results).

A total of 2.89 million reads were mapped at the chosen stringency. Sequencing yield per sample was strongly bimodal, with successful libraries typically producing 50,000–100,000 reads whereas low-yield libraries typically had tens to hundreds of reads. Libraries with fewer than 1,500 total mapped reads were excluded from quantitative analyses of composition. The mean number of reads mapped to reference accessions in the compositionally analyzed samples was 64,166 with a standard deviation of 33,120. Threshold total DNA concentration for strong 12S amplification appeared to be in the range of 5–7 ng/µL, by visual inspection (Fig. 2). That the amplification was related to underlying DNA concentration rather than stochastic or unknown technical factors is indicated by comparing technical replicate libraries (Fig. S15). Among these replicates, library yield was a repeatable characteristic of the sample and reached high values (i.e., counts were limited by library loading rather than by amplification success) when initial DNA yield was 5 ng/µL or higher.

### Taxon skew, rarefaction, technical variation and taxon bias

**Figure 3:**
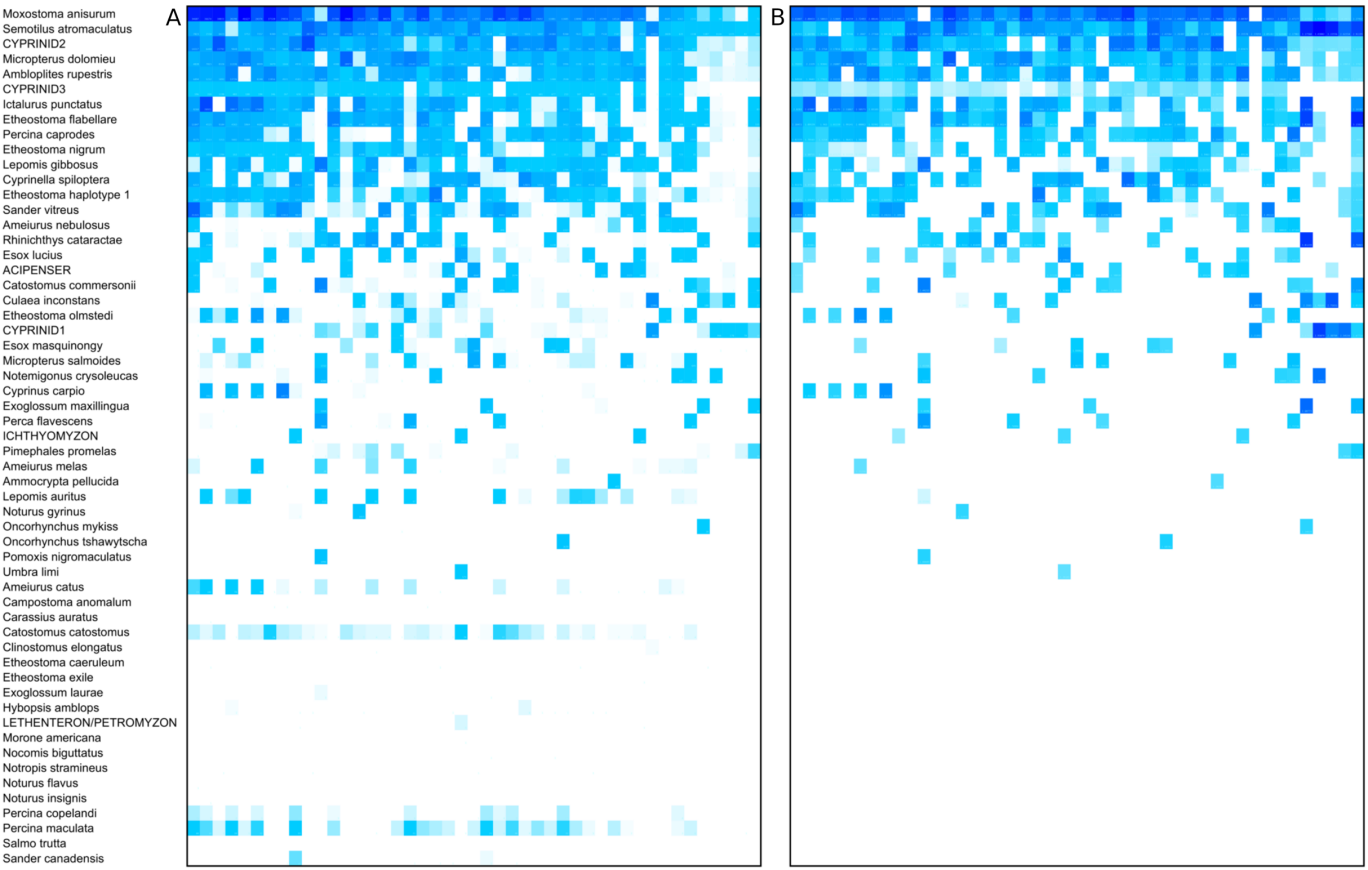
Heat map of taxon abundance by sample. Rows represent detected taxa and columns represent water samples with at least 1,500 12S sequence counts. Samples are arbitrarily sorted by increasing site number and then by sample date, and are unlabeled for image clarity. Color intensity in each cell is scaled by percentile from 0 (no color) to 100% (darkest color) and four potential contaminant species were removed (see Methods). Taxa are ordered by total prevalence above the threshold and then alphabetically. (A) Heat map based on raw counts. (B) Heat map based on scaled log-ratio compositions with a minimum taxon proportion of 0.1% imposed prior to transformation.

The prevalence and relative read abundance of all taxa identified in environmental samples is shown in Fig. 3, before and after censoring cells at a 0.1% threshold (see “Materials and Methods”). Overall taxon abundance was strongly skewed: half of all mapped reads were assigned to the top four bins and 90% were assigned to the top 16 bins. The most prevalent and most abundant taxon by average transformed composition was the *Moxostoma anisurum*–*Hypentelium nigricans* bin. Sequences from the St. Regis River assigned to this bin are presumed to derive only from *M. anisurum* (silver redhorse), one of the most common species observed by capture methods, whereas the known range of *H. nigricans* (northern hogsucker) excludes the St. Regis and nearby watersheds and it was not observed in capture surveys. (The bin is hereafter referred to as *M. anisurum* but *H. nigricans* would be indistinguishable from *M. anisurum* were they co-occur due to their identical 12S references.) Despite this skew, rarefaction curves suggested that most of the richness in these samples can be expected in counts of 10,000 or less (Fig. S16). Additional richness is recovered slowly thereafter, some portion of which is likely due to the accumulation of species via crosstalk reads in addition to genuinely rare taxa.

**Figure 4:**
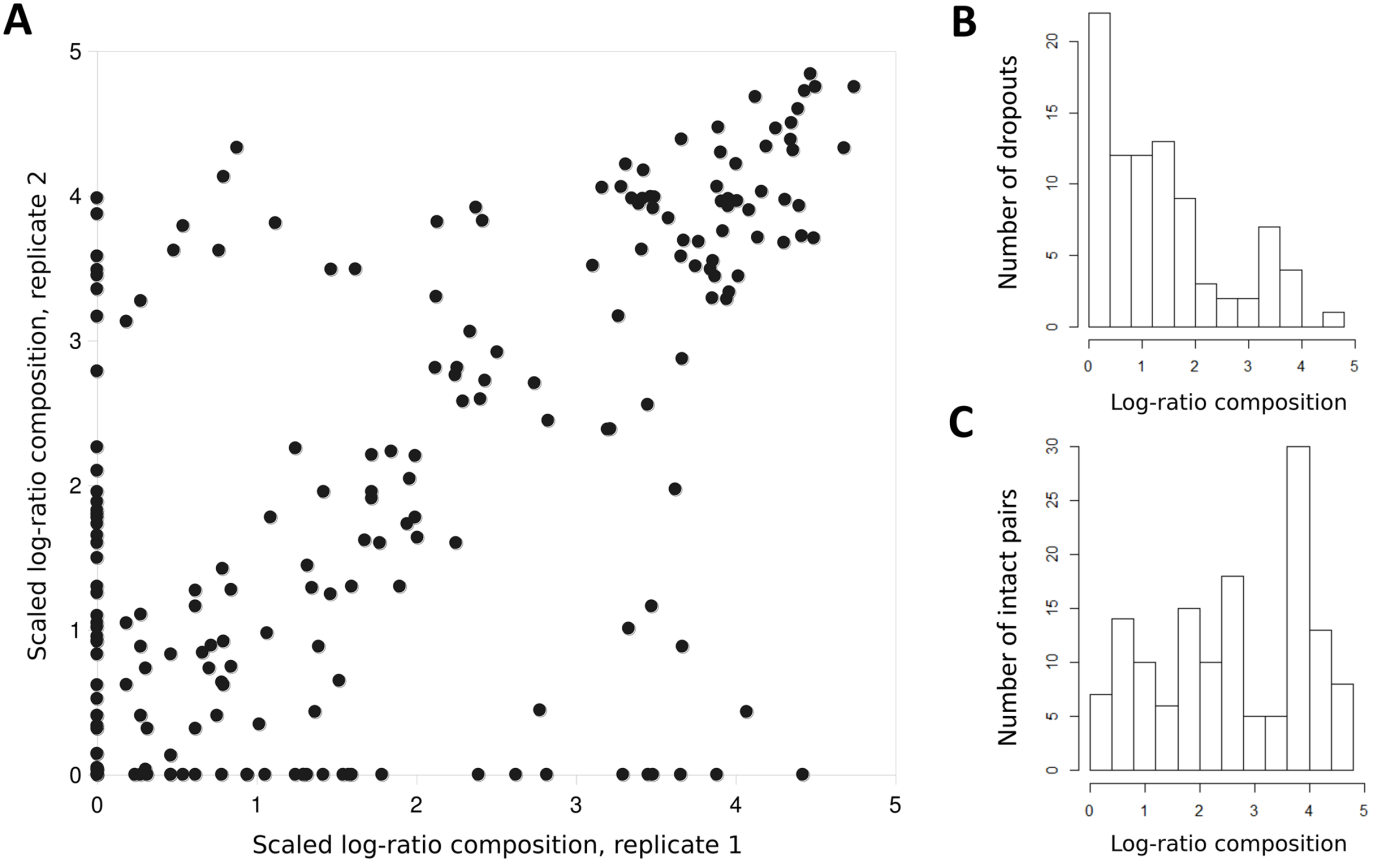
Taxon proportions in technical replicates correlate well overall but exhibit a strong dropout effect. (A) Each point represents scaled log-ratio compositions of individual taxa in two replicates of a single biological sample. Points are pooled across the eight technical replicate pairs (of 12 total) that had at least 1,500 counts per library. (B) Histogram of scaled log-ratio compositions of taxa that were detected in one replicate but not the second. (C) Histogram of mean scaled log-ratio compositions of taxa that were detected in both replicates.

The log-ratio transformed and scaled composition of a taxon in a sample was generally well correlated between replicates, provided some amplification was observed for both (Fig. 4A); the Pearson correlation was *R* = 0.732 (*P* = 6.48E−25) for nonzero pairs. However, complete dropout of a taxon (zero reads) was frequently observed in one replicate of a pair, often but not exclusively at lower relative abundance (Figs. 4B and 4C). This dropout occurred despite the fact that the second-step PCR reaction was performed in triplicate and pooled (see “Methods”).

**Figure 5:**
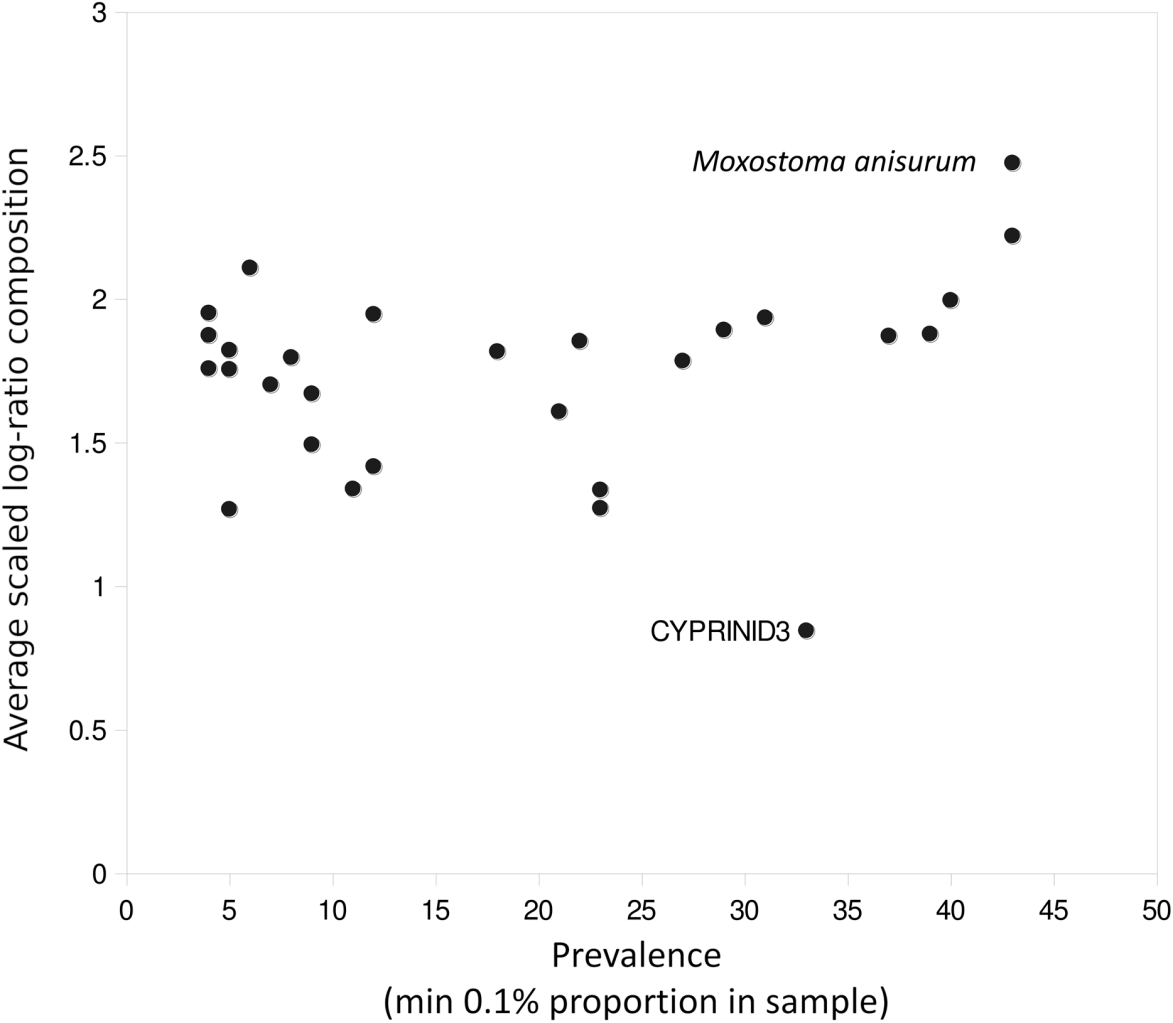
Outlier taxa with respect to average eDNA composition. Scaled log-ratio compositions were averaged across all biological samples with at least 1,500 total counts in which the taxon was present at 0.1% or more. *Moxostoma anisurum* has notably higher average composition than the majority of taxa, whereas the CYPRINID3 bin has notably lower average composition.

**Figure 6:**
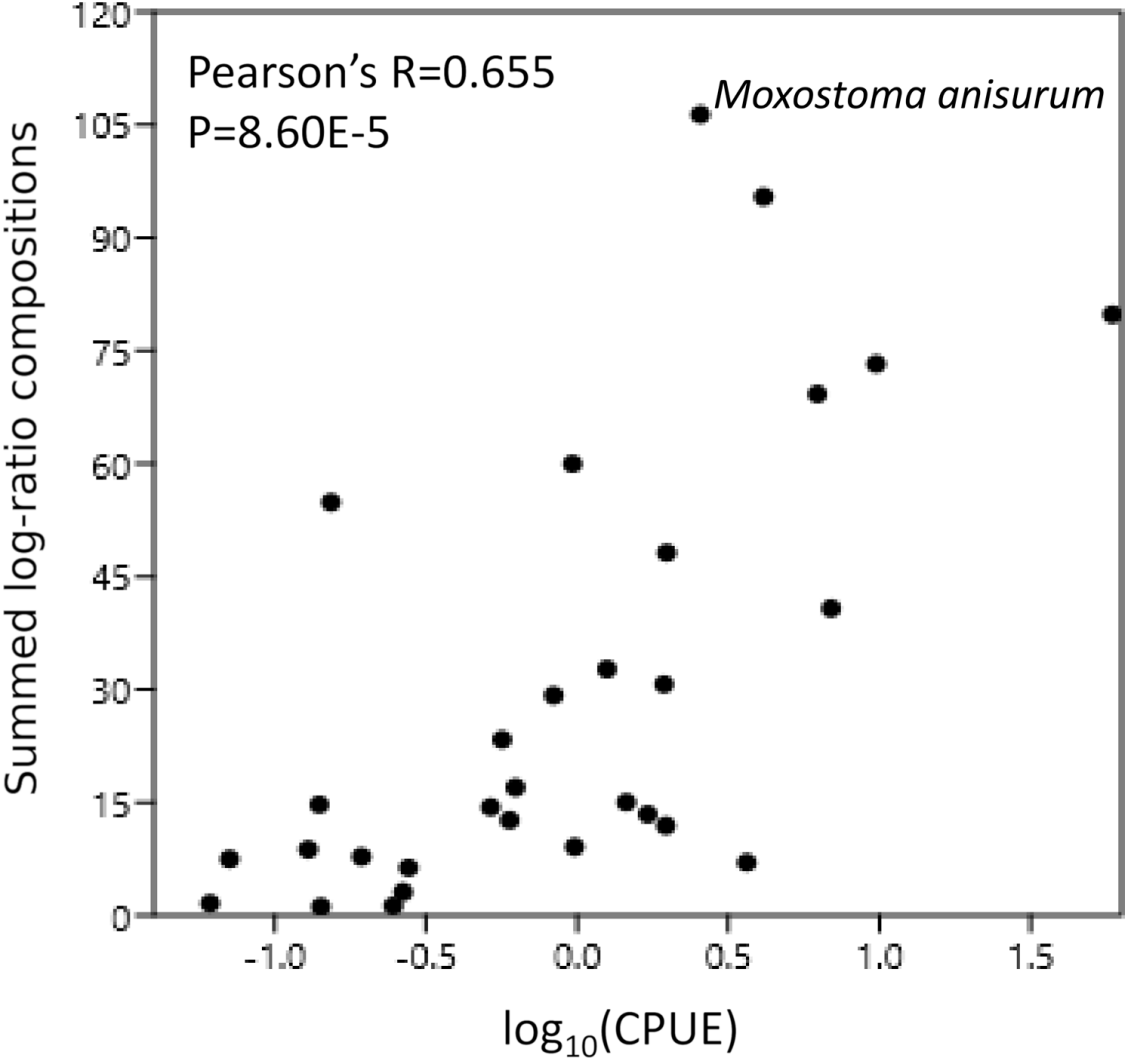
Catch per unit effort (CPUE) correlates with eDNA composition. CPUE was scaled to a total of 100% across all taxa and then log transformed. Scaled log-ratio compositions were summed across all samples in which the taxon was detected at 0.1% or greater

A few taxa tended to have outlier compositions within samples when eDNA composition averaged across all positive samples was plotted against prevalence (Fig. 5). *M. anisurum* in particular had higher average composition in samples than other common taxa as well as relative to CPUE (see below and Fig. 6), suggesting a positive amplification bias. In contrast, the CYPRINID3 bin was consistently rare in the samples in which it was detected, which could be due to negative amplification bias but we consider a “shadow effect” more likely. That is, a low rate of misassignment of reads from a common taxon to a rare or absent taxon would cause the latter to have both high prevalence and low abundance, as well as create a strong correlation with the true source. Indeed, CYPRINID3 was never detected above threshold level in the absence of CYPRINID2, and the two bins had a Pearson correlation coefficient of 0.970 when both were detected above threshold (Fig. S17). While some samples had CYPRINID3 compositions higher than what might be expected based on misassignment of CYRPINID2 reads alone, indicating that this bin was legitimately present in these cases, we elected to remove CYPRINID3 from the quantitative comparisons below.

### Comparison of eDNA composition with CPUE

CPUE were available for 31 of the 34 taxonomic bins in Table 1; for multispecies bins, we summed CPUE across all observed species assigned to those bins. After removing the CYPRINID3 bin and taxa for which CPUE were not available (see “Methods”), the summed eDNA compositions were significantly correlated with log(CPUE) (Pearson’s *R* = 0.655, *P* = 8.60E−5; Fig. 6). Nonparametric correlations were also significant (Fig. 6).

### Within-watershed comparisons of eDNA composition

**Figure 7:**
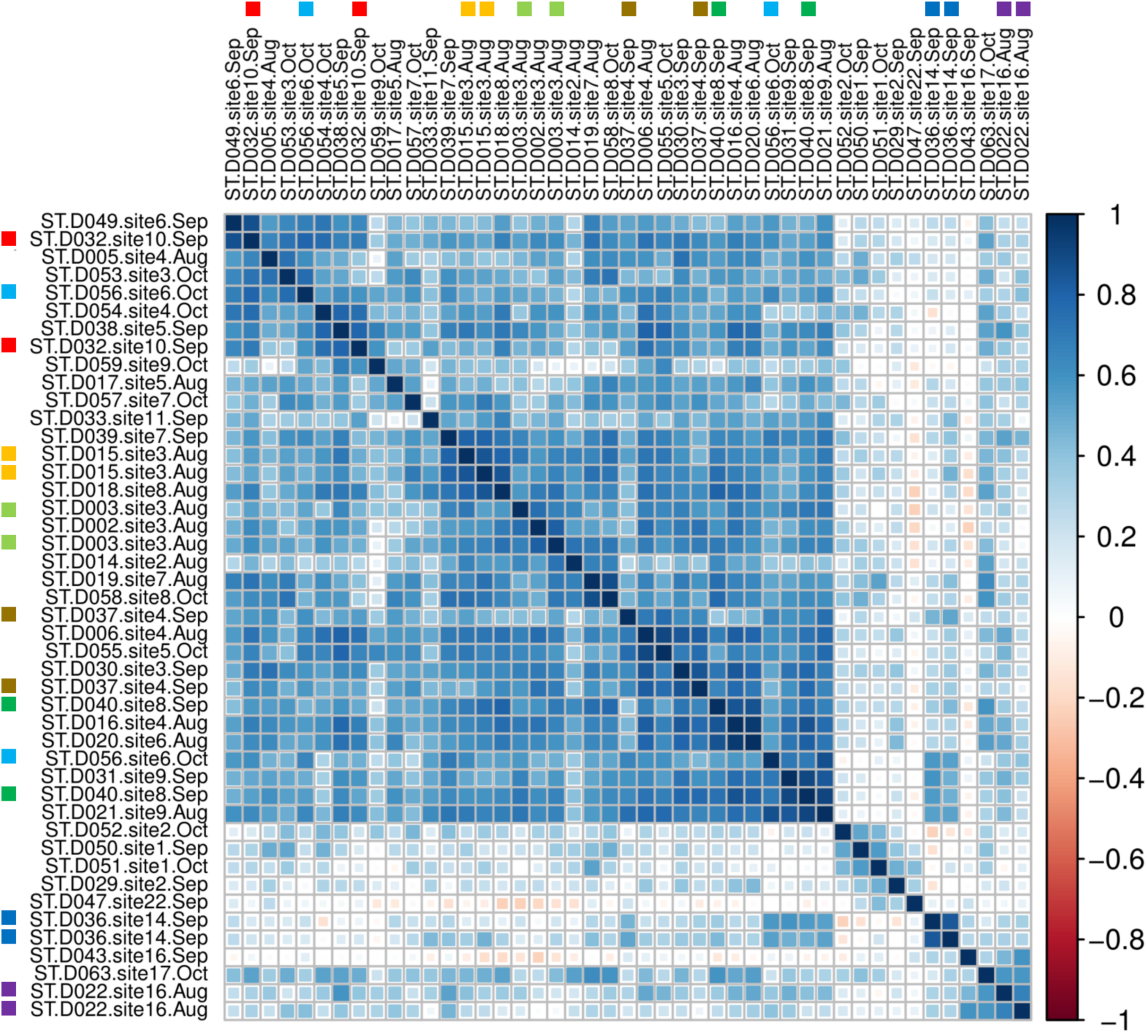
Among-sample similarity in eDNA composition. Color scale represents pairwise values of Spearman’s rank correlation coefficient and the order of samples is based on clustering by Ward’s method. Technical replicate pairs are marked by matching colored boxes. Taxa with a prevalence of less than four samples at a minimum abundance of 0.1% (prior to transformation) were excluded.

A sample-level correlogram based on Spearman’s *r* and clustered with Ward’s method (Fig. 7) showed that most lower-reach samples above and below the Hogansburg Dam, NY, USA were relatively similar, whereas the few successful headwater and middle-reach libraries were well differentiated. Biological replicates (collected from the same site on the same or different dates) and technical replicates (replicate sequencing libraries created from the same sample DNA and marked with colored squares in Fig. 7) sometimes clustered near each other but not exclusively so, presumably due to the overall similarity of eDNA compositions in the lower reaches and noise arising from the dropout effect noted previously. Sites 14 and 16 in the upper portions of the river clustered together. Site 22, located at the outflow of a series of small lakes, clustered near samples from sites 1 and 2, which are immediately upstream of the St. Lawrence River.

**Figure 8:**
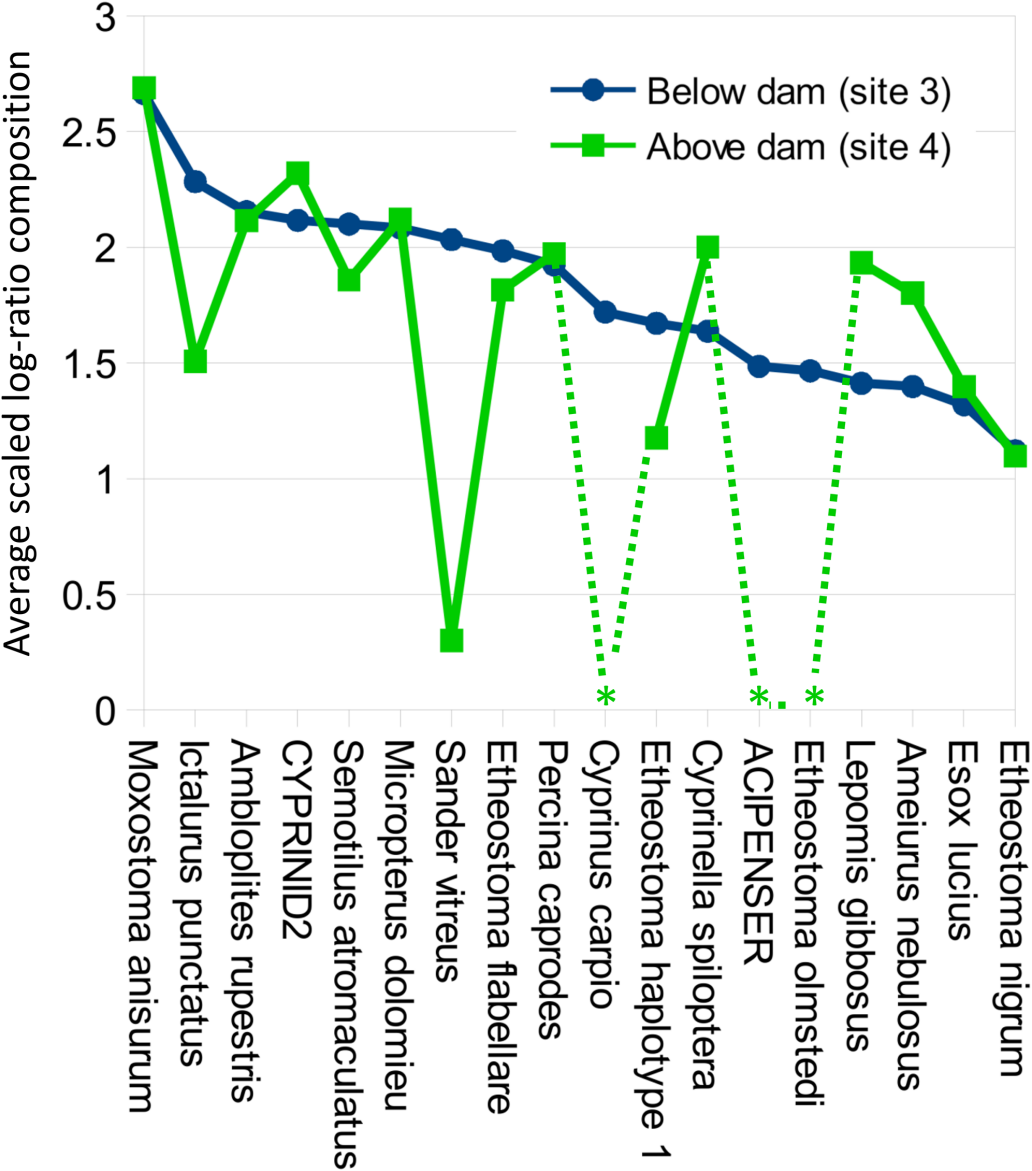
Per-taxon changes in average eDNA composition above and below the Hogansburg Dam, NY, USA. Averages are of five biological replicates at each site, and only taxa detected in at least two of the five sampling events at a single site were included. Asterisks indicate zero detections above the 0.1% threshold for that taxon at the corresponding site. Taxa are sorted by average log-ratio composition at site 3 (below the dam), in descending order.

**Figure 9:**
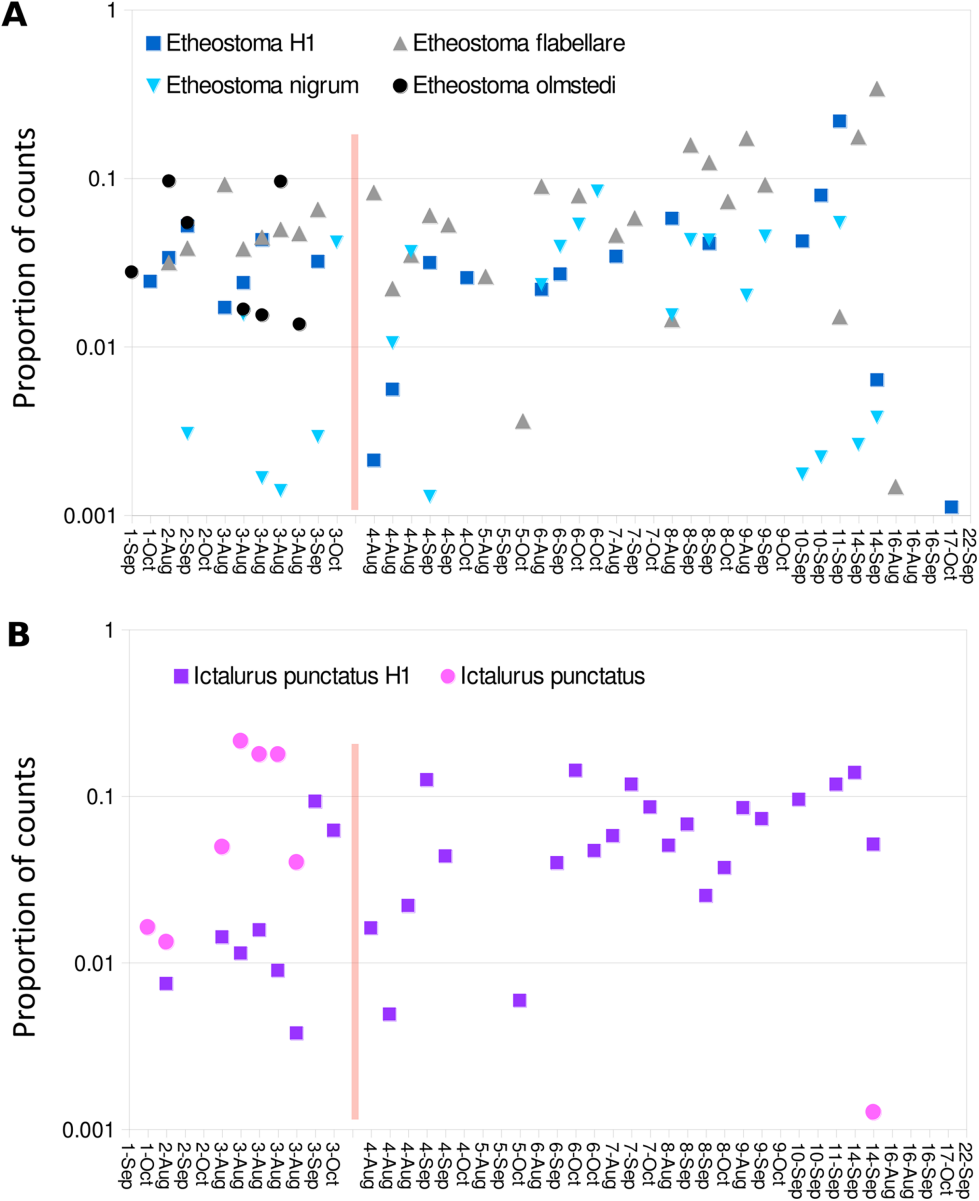
Haplotype distribution for two groups of taxa show shifts coincident with the Hogansburg Dam, NY, USA. Samples with greater than 1,500 total counts are shown ordered from downstream to upstream, by sampling date, with the dam location marked by a red line. For samples with technical replicates, only the replicate with the highest total counts is shown. Values are shown as proportion of counts for equivalence of scale across samples. (A) Shows four *Etheostoma* bins, including *Etheostoma* haplotype 1 (*Etheostoma* H1) which is of uncertain taxonomy but is closest by edit distance to *E. nigrum* (see text for details). (B) Shows aggregate values obtained for reference sequences of *Ictalurus punctatus* and for the novel haplotype attributed to that species (*Ictalurus punctatus* H1).

Despite the overall compositional similarity in the vicinity of the Hogansburg Dam, NY, USA suggested by Fig. 7, changes in eDNA compositions between sites 3 and 4 (on either side of the dam prior to removal of the structure (Fig. 1)) suggest an impact on particular species (Fig. 8). Channel catfish (*I. punctatus*), common carp (*Cyprinus carpio*), sturgeon (bin ACIPENSER) and walleye (*Sander vitreus*) were absent or exhibited sharp dips in eDNA composition immediately above the dam. Taxa that had higher eDNA compositions above the dam included brown bullhead (*Amerius nebulosus*), spotfin shiner (*Cyprinella spiloptera*) and longnose dace (*Rhinichthys cataractae*), but these differences appear smaller in magnitude. While sample sizes were not large enough to statistically test between-site differences in eDNA abundance for each taxonomic bin, the asymmetry in the magnitude of differences is consistent with the flow of eDNA downstream from site 4 to site 3. The impact of the dam is also revealed by discontinuity in the distribution of some of the alternative haplotypes identified for some taxa (Fig. 9). The reference *I. punctatus* haplotype occurred almost exclusively below the dam, whereas the alternative haplotype was common both above and below the dam. The reference *E. olmstedi* haplotype also occurred only below the dam, whereas the reference *E. nigrum* haplotype was generally rare below the dam and abundant above it. The taxonomic source of *Etheostoma* haplotype 1 remains to be determined by voucher sequencing but it is a single edit distance from the reference *E. nigrum* (Fig. S11) and both were frequently detected together (Fig. 9).

## Discussion

In order to validate eDNA monitoring as a management tool for the St. Regis River, we investigated the diversity and resolution of detected fish taxa and compared our inferences against a body of traditional survey data. This pilot analysis was accomplished with a simple sampling technology and a sampling scheme that, with a single MiSeq chip, could provide information on temporal and spatial variation, sources of technical noise, and potential levels of contamination. Overall, this strategy was successful in addressing the questions we had posed and verified that 12S sequencing is a cost-effective approach with good taxonomic resolution and good concordance with traditional surveys in aggregate.

### How much filtrate is needed?

We identified a narrow threshold of eDNA concentration above which sequencing library yield rapidly increased, such that samples with initial DNA yields less than 5 ng/µL (1.5 µg in total) had low success rates in our hands. Adjusting elution or dilution steps, or using larger reaction inputs, or both, would likely improve library yield without altering the field acquisition procedure. While the 300 mL volumes processed here were relatively small, other studies have used similar or even smaller volumes (Li et al., 2018) and the simplicity of the approach allowed up to eleven sites to be visited in a single day (Supplemental File S1). Nonetheless, in headwater sites, a larger filtrate will likely be needed. Interestingly, library yield declined at the highest sample DNA concentration (Fig. 3; Fig. S15), which corresponds to the outlier value marked for August in Fig. S14. While it is not possible to generalize from this single observation, a high DNA concentration could indicate enrichment of bacterial or other DNA source that is depauperate in fish eDNA (Cornman et al., 2018).

### When should sampling be performed?

We obtained strong libraries from August and September samples in the lower sections of the river (Fig. S14; Supplemental File S1), whereas there was a substantial decline in eDNA abundance in October. The decline in DNA yield in the fall is consistent with other studies (Buxton et al., 2017; Buxton, Groombridge & Griffiths, 2018) and the expected metabolic decline of many resident species. Historical data indicate that flows in the St. Regis are relatively low and stable during August and September, which could be advantageous for sampling with regards to access and consistency. Indeed, concentrations of fish eDNA in the St. Regis could well peak in late summer given the preceding months of higher metabolism and population growth coupled with lower flows, but seasonal trends remain to be empirically demonstrated. Early-season eDNA compositions might also be intrinsically more variable due to spawning behavior (Erickson et al., 2016; Tillotson et al., 2018), peak flows, and differing metabolic curves among species as habitats warm. On the other hand, reproduction and seasonal movements are often important variables in fisheries management that, for most species, would not be reflected in late summer sampling.

### How should taxa be binned?

Of 132 freshwater species identified on the state checklist, we found complete or partial 12S reference sequences for 110, or 83.3%. Eleven multispecies bins were needed to limit estimated per-taxon error rates to 5% (Fig. S3), which for generality assumed all checklist taxa are possible. Further narrowing of these bins may well be possible for specific sites based on other independent data, such as habitat requirements, catch data, or high-resolution range maps. For example, the only species of the CYPRINID3 bin that was actually observed in the St. Regis River capture data was *Notropis hudsonius* (Table 1). The common approach of aggregating counts at more inclusive taxonomic ranks (e.g., genus or family) to accommodate assignment uncertainty would typically strip much information from the data set. This is particularly true for cyprinid minnows, as they are highly radiated and have unsettled taxonomies (Stout et al., 2016), such that species resolution is challenging to achieve. Tolerating low estimated error rates and using explicit binning is in our opinion more useful than the blanket approach, and in many cases there may be no management need to differentiate cyprinids further. On the other hand, low rates of misassignment of very common taxa could swamp the counts attributed to much rarer relatives, as appeared to be the case with the CYPRINID2 and CYPRINID3 bins in our data.

### What taxa were detected and in what proportions?

Several studies have compared eDNA detections with traditional fish survey methods and show convergence toward very similar sets of taxa identified in aggregate, that is, for significant survey efforts spanning space and time (Thomsen et al., 2016; Hänfling et al., 2016; Pont et al., 2018; Gillet et al., 2018; Goutte et al., 2020). In the St. Regis, few expected taxa were recalcitrant to eDNA (Table 1), but four missing taxa were noted for which primer-site divergence could be excluded as a cause. Other work (Li et al., 2018; Evans et al., 2016; Farley et al., 2018; Weldon et al., 2020) suggests these species should have been detectable by eDNA, so we assume their absence in our eDNA survey was stochastic and would be overcome with additional sampling.

The distribution of total reads in the data set was strongly skewed, such that the top four taxonomic bins accounted for over half of all sequences (*M. anisurum*, *S. atromaculatus*, CYPRINID2 and *M. dolomieu*) and the top sixteen bins accounted for over 90% (Supplemental File S5). A number of detections were based on very few reads, such that the possibility of contamination could not be meaningfully evaluated for these taxa and no quantitative analysis could be performed. While primer mismatch does not seem to explain non-detections in general (Fig. S12), amplification bias is suggested by the high *M. anisurum* compositions recovered (Fig. 5). While amplification biases can be evaluated directly by mock community analysis, we believe it is advisable to avoid combining high-concentration control samples with environmental samples in the same sequencing run due to higher rates of sample crosstalk (Olds et al., 2016) and increased risk of sample contamination by abundant PCR product.

Based on our results, a substantial increase in throughput would be needed for quantitative monitoring of taxa that had low recovery at the 12S locus, or alternative barcode loci could be explored. Fortunately, methods have been described for amplicon sequencing on the HiSeq platform (De Muinck et al., 2017; Holm et al., 2019) that could increase yield by more than an order of magnitude if successfully applied to this use case.

We observed eDNA compositions to be reasonably stable between technical replicates overall, but with a surprisingly frequent dropout of individual taxa from only one replicate of a pair (Fig. 4). This high dropout rate occurred despite the pooling of triplicate PCRs when Illumina-specific adapters were appended. While dropout was more likely at lower compositions, dropout at higher compositions remained a notable fraction of the total. We suspect this observation is related to that of O’Donnell et al. (2016), in which large variation among technical replicates could be attributed to the multiplex adapters themselves. In that study, pooling replicate preamplification PCRs prior to adding multiplex adapters eliminated the effect.

### Were detections concordant with traditional surveys?

In addition to a high overlap in detected species, we also found log-ratio eDNA compositions to be relatively well correlated in aggregate with log-transformed CPUE (Pearson’s *R* = 0.655, Fig. 6), the latter being a common measure of taxon abundance in fisheries data. This correlation was assessed at the level of the whole watershed and was similar in strength for Spearman’s rank correlation coefficient as well. Evans et al. (2016) found generally strong correlations between taxon biomass and untransformed 12S read counts in a mesocosm study. Of course, mesocosms by design exclude many of the complicating factors that occur in watersheds such as fish movement, water flow, and environmental heterogeneity (e.g., of sediments, microbial activity, and water chemistry). Pont et al. (2018) found similarly strong correlations between eDNA and CPUE at individual sites along the Rhone River for which long-term data were available. It is possible that weighting CPUE by catch biomass (Evans et al., 2016) or allometric factors (Yates et al., 2020), rather than counts, might further improve correlations with eDNA.

### Did eDNA profiles reflect major watershed features?

Multivariate clustering (Fig. 7) illustrated the distinctiveness of headwater samples and sites influenced by other waterways (i.e., immediately upstream of the St. Lawrence or immediately downstream of headwater ponds). Both replicate pairs from upstream samples clustered as a set, indicating that technical noise was less than the scale of community divergence (Fig. 7). However, the lower river was largely unstructured in terms of eDNA composition, such that replicates did not always cluster together. Future sampling can likely be reduced in this region for most applications.

A key finding of this study was the apparent impact of the former Hogansburg Dam, NY, USA on eDNA compositions for particular taxa (Fig. 8) as well as on genotypic distributions of channel catfish (Fig. 9) in particular but also *Etheostoma* to some extent. Other studies have also documented dams as barriers to gene flow for channel catfish (Sotola et al., 2017). We did not systematically test genotypic distributions relative to the dam for all taxa as 12S polymorphism in the reference database was limited. However, we did not observe obvious genotypic structuring in other common, genetically polymorphic species such as largemouth bass (*Micropterus salmoides*) and creek chub (*Semotilus atromaculatus*). It would be informative to repeat the analysis to confirm whether these instances of compositional or genotypic structure have dissipated over the several years since dam removal.

### Bioinformatic approaches to evaluating alignment-based taxonomy

While the mapping of reads to references is a common approach to high-throughput taxonomic inference, particularly of relatively stable and catalogued communities, validation and calibration remain necessary to assess potential errors. In this study, we used simulated data under the assumption that the reference database closely matched the actual St. Regis fish community, which seemed reasonable given that the region is well studied and multiple unique reference sequences were available for many species. Nonetheless, we identified several types of discordance among reads mapped to the reference database. We expect that most such cases of ambiguity or discordance can be clarified with targeted voucher collection and sequencing, in an iterative manner, and indeed may bring novel biodiversity to light.

Mapping stringency is often based on the proportion of bases that match but can also encompass other alignment characteristics that are reported by a given alignment package. Two common alignment specifications are the BTOP format reported by BLAST (National Center for Biotechnology Information, 2008) and the CIGAR and MDZ strings used by the SAM specification (Li et al., 2009). In this study, we customized our mapping stringency by considering the number of mismatches (*M*), the number of gap positions (*G*) and the truncation (*L*) of the alignment below the maximum possible length as distinct variables. While we are not aware of other studies that have used this particular scoring approach, alignment programs that perform local mapping, such as bowtie2 (Langmead & Salzberg, 2012), optimize a very similar suite of variables using user-specified scoring weights. We do not claim our scoring approach is ideal for all data sets or simple to implement, but it more explicitly defines an acceptable alignment. For simulated data, read counts were most sensitive to changes in L, which limits the number of reference bases that are left unaligned. The gap threshold G had little importance in the simulated data because indels were modeled at the low rate they typically occur in sequencing by synthesis. However, gaps were clearly an important variable in real data (Fig. S5), presumably because the reference database contained indels relative to conspecifics occurring in our environment. This observation is unsurprising in retrospect, as reference sequences generated by the Sanger methodology are susceptible to indels arising from poorly resolved fluorescence peaks and often are based on single coverage, and it illustrates the benefit of having separate alignment thresholds for gaps versus mismatches.

In contrast to reference-based assessment of taxonomic compositions, alternative de novo approaches employ explicit models of variation in sequence reads arising during PCR amplification to generate “de-noised” cluster representatives or “exact sequence variants”. These algorithms seek to discriminate the underlying biological templates from the messy amplicons that are propagated during PCR and sequencing (reviewed in Nearing et al. (2018)) and are particularly important for high-diversity microbial studies that often lack alternative means of verifying novel haplotypes. The denoised OTUs can then be taxonomically assigned by methods such as kmer-based classifiers (Wang et al., 2007; Edgar, 2016) or phylogenetic classifiers (Munch et al., 2008; Matsen, Kodner & Armbrust, 2010). Denoising methods do not typically produce alignments for further evaluation, however. An approach similar to what we used here would still be needed to review the pattern of variation within clusters for potential artifacts, which was our primary purpose in adopting it. Nonetheless, reference-alignment approaches are not incompatible with de novo clustering and both could be implemented in the same study, that is, by denoising the read pool prior to mapping or by denoising pools of reads that map to the same reference or taxon.

### Future Directions

To reliably implement eDNA monitoring in the upper reaches of the St. Regis and similar watersheds, it will be important to confirm that the threshold for initial DNA extracts of 5 ng/µL approximately holds for library preparation and can be achieved by a simple linear increase in filtered water volumes or laboratory adjustments to increase DNA input in the preamplification reaction. It will also be important to compare these eDNA compositions with those from spring or early summer samples. Our results also suggest that additional voucher sequencing is needed to generate the most representative database.

In the study, we exclusively examined the 12S locus, which has been the most productive locus in several multilocus comparisons (Shaw et al., 2016; Hänfling et al., 2016; Gillet et al., 2018) but not all (Li et al., 2018). A large majority of expected taxa were detected with 12S reads in aggregate in this environment (Table 1), to the limit of marker resolution, with only modest levels of off-target amplification (Supplemental File S13). The small size of the 12S locus also favors scaling throughput to larger platforms and 12S read abundance has shown good agreement with independent measures of source population size as discussed above. Nonetheless, we believe additional loci should still be investigated, particularly when sequencing pooled samples at high depth for validation purposes. Multilocus confirmation of taxa will often be necessary when independent records are scarce (Evans et al., 2017).

## Conclusion

Overall, rapid sampling achieved a cost-effective assessment of eDNA distributions in a watershed and provided important criteria for determining future sampling effort. Furthermore, 12S sequencing achieved relatively high taxonomic resolution using a binning approach based on expected error and assuming that modest levels of uncertainty can be tolerated. While eDNA compositions were strongly skewed, they were nonetheless significantly correlated with log(CPUE) from capture surveys and most taxa observed by traditional capture were detected by eDNA. Although the middle and lower portions of the St. Regis had relatively similar eDNA compositions, sites near the St. Lawrence and in the headwaters were distinct. The effect of the former Hogansburg Dam, NY, USA was apparent in the abundance or haplotype distributions of some taxa. We suggest that eDNA compositions can be evaluated with minimal investment in sampling technology at the outset and encourage exploration of aligned reads with standard bioinformatic tools as a means of evaluating the concordance of reference sequences with those obtained from the local environment.

## Supplemental Information

10.7717/peerj.10539/supp-1 Supplemental Information 1 Diagram of steps in the bioinformatic workflow used to produce the primary taxon counts table

10.7717/peerj.10539/supp-2 Supplemental Information 2 Distribution of mismatch rates for largemouth bass (*Micropterus salmoides*), smallmouth bass (*Micropterus dolomieu*), and rock bass (*Ambloplites rupestris*) from which error rates were modeled Proportion of mismatches at each reference position from 10-90, corresponding to sequencing cycle and not an alignment position. Proportions are calculated from unique reads only, dereplicated at 100%, not total aligned reads in the data set. The edges of the references were not used in the estimation because edge effects (e.g. skips, residual adapter matches, reference length variation) are reference specific and not generalizable sources of error. However, the fitted relationship applies to the entire reference in the simulated data.

10.7717/peerj.10539/supp-3 Supplemental Information 3 Binning of taxa based on identical reference sequences and estimated error rates between similar references Networks represent undirected adjacency data (misassignment rates between species pairs). Taxa with error rates greater than 5% were included, along with the taxa that they are conflated with (see Methods for details).

10.7717/peerj.10539/supp-4 Supplemental Information 4 Number of simulated reads mapped to references under various parameter combinations The horizontal axis lists iterations of the three parameter values investigated, in the form “G_L_M”. For example, “1_3_3” corresponds to G = 1, L = 3, and M = 3. G represents the number of gap positions in the alignment, L is the difference between the reference sequence length and the aligned sequence length, and M is the number of mismatched positions. See text for details. The vertical axis indicates the number of simulated reads mapped.

10.7717/peerj.10539/supp-5 Supplemental Information 5 Number of unique reads from environmental samples mapped to references under different values of G, the number of gap positions in the alignment Axes are log-scaled and indicate the number of simulated reads mapped to source taxa under the thresholds indicated.

10.7717/peerj.10539/supp-6 Supplemental Information 6 Inference of contamination and demultiplex error from negative control samples (A) Scatterplot of total counts for each taxon in biological samples versus negative controls. Dashed line indicates a rate of 1 in 1,000 counts in the combined negative controls relative to the combined biological samples, for illustration purposes only. (B) A heatmap of counts of each taxon in each negative control sample, with darker coloring indicating greater abundance. Taxa and samples presumed to be contaminated are marked by dots. The starred negative-control sample was reported to be potentially contaminated by the field crew.

10.7717/peerj.10539/supp-7 Supplemental Information 7 Correlation matrix of initial taxon counts (transformed and scaled), prior to validation Only taxa present in at least four samples at a minimum composition of 0.1% were included in this comparison. Spearman’s nonparametric correlation coefficient (r) was specified and the order of samples clustered by Ward’s method with corrplot (Wei and Simko 2017). Yellow boxes highlight pairs of taxa with high correlations coefficients that are discussed in the text.

10.7717/peerj.10539/supp-8 Supplemental Information 8 Haplotype intermediate between *Percina caprodes* and *Percina macrocephala* aligned with references The first sequence is a representative environmental sequence drawn from the pool mapping to *P. caprodes* and *P. macrocephala*. Dots indicate bases that do not vary from the corresponding position in the first sequence. Variant sites are marked by arrows. The environmental sequence is identical to *P. rex*, which is an endemic of Virgina and not present in New York. The environmental sequence is an edit distance of two from both *P. caprodes* and *P. macrocephala*.

10.7717/peerj.10539/supp-9 Supplemental Information 9 Alignment of a single, high-abundance haplotype with reference sequences of five cyprinid species Dots indicate bases that do not vary from the corresponding position in the first sequence. Reference sequences are invariant within species. The environmental sequence was the cluster representative reported by vsearch after clustering the pool of reads mapping to taxonomic bins with correlated counts (see text for details).

10.7717/peerj.10539/supp-10 Supplemental Information 10 Visualization of haplotype variation reflected by mismatch distribution Mismatch distributions are shown for references with at least 100 mapped reads. Each set of four points represents the proportion of mapped reads with 0 to 3 mismatches, respectively. Most accessions have a single peak, whereas multimodal curves suggest unique haplotypes aligning to the same reference (labelled by accession number and reference taxon). Upper panels illustrate read ‘pileup’ relative to the reference accession, with variant sites colored pink.

10.7717/peerj.10539/supp-11 Supplemental Information 11 Alternative haplotypes for four taxa Taxa correspond to accessions from Figure S11 as well as additional relevant species obtained by BLASTN alignment to NCBI’s nucleotide database. Dots represent bases that do not differ from the first listed sequence in each alignment. For each alignment, haplotypes that are distinct from existing references are indicated.

10.7717/peerj.10539/supp-12 Supplemental Information 12 Alignment of 12S primer regions from whole mitochondrial genomes for undetected taxa No mismatches occur in the primer regions (forward and reverse complement primer sequences are indicated in the bottom two rows).

10.7717/peerj.10539/supp-13 Supplemental Information 13 Pie chart of the inferred sources of unmapped reads by taxonomic class The majority of unmapped reads are from ray-finned fishes and are presumed to be low-quality reads deriving from the same species as mapped reads. Taxonomic assignments inferred by the lowest common ancestor (LCA) method as described in the Methods. See File S5 for detailed LCA results.

10.7717/peerj.10539/supp-14 Supplemental Information 14 Historical flows of the St. Regis River by month The primary axis indicates the discharge in cubic feet per second (cfs) since 1917, averaged by month. The secondary axis represents the dispersion around those means, measured as the coefficient of variation (CV). Values are based on an online database of historical records for U.S. Geological Survey water gauge 105838 at Brasher Center, New York. eDNA concentrations obtained by this study are illustrated by violin plot in the inset, by month. Boxes within each violin plot denote quartile values. The outlier eDNA concentration in August was the highest value obtained (20.3 ng/μL) but had a relatively low library yield in both technical replicates (see Figure 3 and text).

10.7717/peerj.10539/supp-15 Supplemental Information 15 Library yield of technical replicate pairs Twelve technical replicate pairs were performed, represented as dots connected by vertical lines. Each technical replicate had the same source DNA concentration. The vertical axis represents total number of mapped reads (unmapped reads do not contribute to library size). The two replicate pairs with the largest variance are closest to the 5 ng/uL concentration marked on the figure.

10.7717/peerj.10539/supp-16 Supplemental Information 16 Rarefaction curves for all samples included in the compositional analysis (i.e., with greater than 1,500 mapped reads). Red line denotes 1,500 read counts. Taxa are the final species and multispecies bins grouped as described in the text.

10.7717/peerj.10539/supp-17 Supplemental Information 17 Taxon correlations across biological samples after revision of the reference database (A) Correlation matrix of all species and multispecies bins. (B) Correlation between CYPRINID2 and CYPRINID3 compositions for samples in which both were detected.

10.7717/peerj.10539/supp-18 Supplemental Information 18 Sample metadata Sample names, NCBI accessions, coordinates, collection dates, and other sample metadata.

10.7717/peerj.10539/supp-19 Supplemental Information 19 Phylogenetic curation of discovered reference accessions A neighbor-joining tree used to identify phylogenetically discordant accessions for exclusion from the reference database. Accessions marked “deleted” were considered to be potentially misidentified during submission to NCBI and removed from the reference database.

10.7717/peerj.10539/supp-20 Supplemental Information 20 Code used to parse SAM-formatted alignments based on gap, length, and mismatch criteria The script is written as pseudocode, that is, it cannot be executed without modification by a knowledgeable user for a specific computer system. The pseudocode only documents the procedure used and is not software for consumption.

10.7717/peerj.10539/supp-21 Supplemental Information 21 Final reference database

10.7717/peerj.10539/supp-22 Supplemental Information 22 Counts tables for different classes of sample and analysis Each sheet of the spreadsheet contains samples selected for specific analyses, for example, duplicates, negative controls, and biological samples above threshold library sizes. Sample names correspond to those in File S1, but with the unique MiSeq sample ID appended as well. Technical duplicates are marked by the text “dup” in the sample name. Raw counts are provided, but were censored, transformed, or scaled as described in the text depending on the analysis.

10.7717/peerj.10539/supp-23 Supplemental Information 23 Lowest common ancestor analysis of unmapped reads Reads were clustered with vsearch and cluster representative searched against the nt database. Each cluster was assigned the basal taxonomy of the top matches, if any, selected as described in the methods. This spreadsheet lists the taxonomic assignment and alignment statistics of the top hits.

10.7717/peerj.10539/supp-24 Supplemental Information 24 Analysis of variance of eDNA concentration by month The output table generated by PAST3, including pairwise post hoc comparisons.

## Additional Information and Declarations

### Field Study Permissions

The following information was supplied relating to field study approvals (i.e., approving body and any reference numbers): All field work was done as employee duty under the authority of the U.S. Geological Survey, U.S. Department of Interior. No specific permit is required to collect water samples for environmental DNA analysis in the State of New York. Fish observation records were aggregated from existing data sources and thus are not specifically attributable to this study.

### DNA Deposition

The following information was supplied regarding the deposition of DNA sequences: FASTQ reads for biological samples are available at NCBI: PRJNA646929. In accordance with U.S. Geological Survey policy, data pertaining to this project is available at an agency-approved repository: Cornman, R.S., McKenna Jr, J.E., and Fike, J.M., 2020, Taxonomic composition of environmental DNA acquired by filtration from the St. Regis River, New York: U.S. Geological Survey data release, DOI 10.5066/P9EEOAZK.

### Data Availability

The following information was supplied regarding data availability: Raw sequence data are available at NCBI: PRJNA646929. Analysis code and taxonomic count data are available in Supplemental Files.
