# Supplementary figures and images for: Composition and distribution of fish environmental DNA in an Adirondack watershed
Source: PeerJ. 2021 Feb 26;9:e10539. doi: 10.7717/peerj.10539 (PMC7919543; doi:10.7717/peerj.10539)

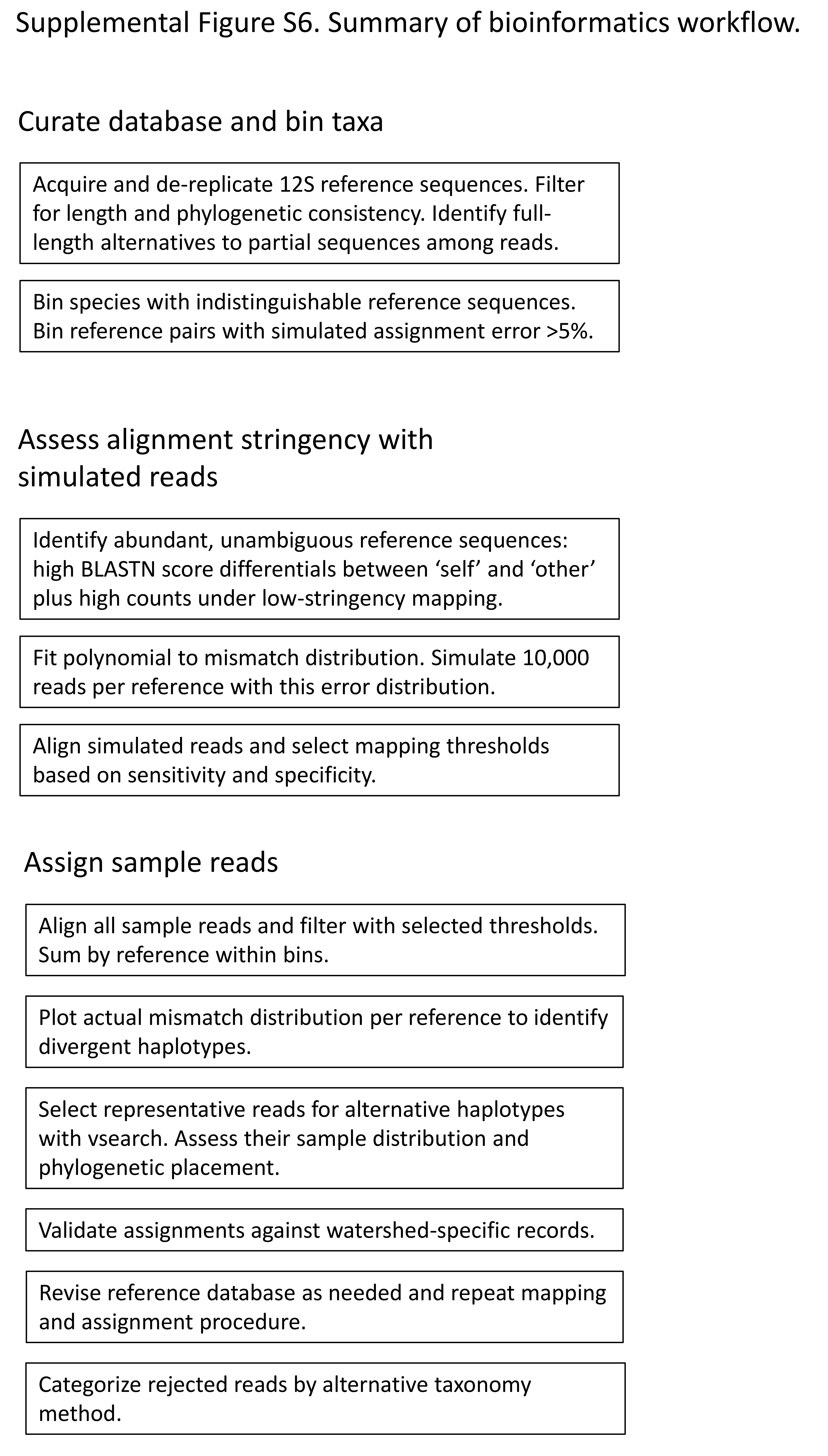

Supplement: Supplemental Information 1 [file peerj-09-10539-s001.png]

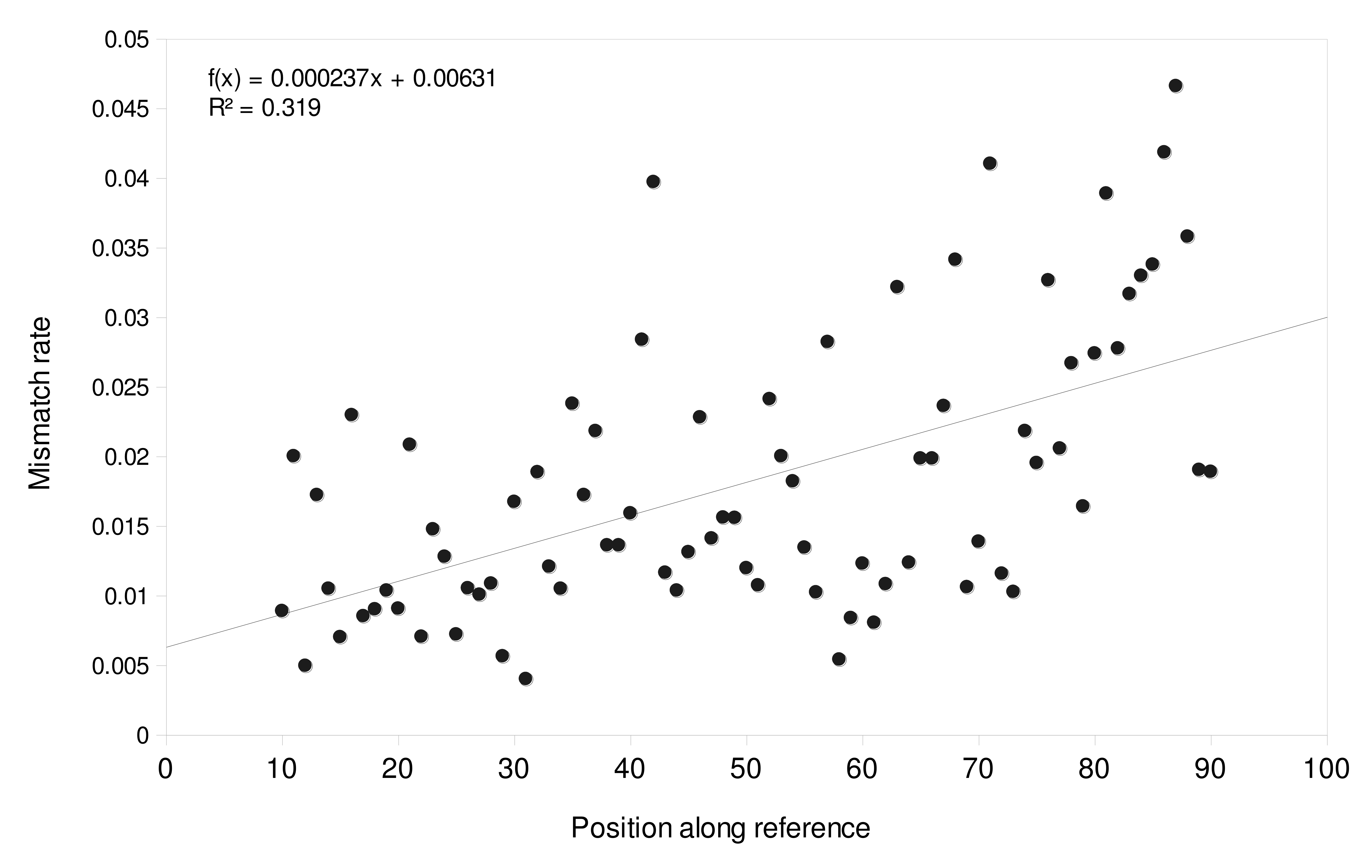

Supplement: Supplemental Information 2 — Proportion of mismatches at each reference position from 10-90, corresponding to sequencing cycle and not an alignment position. Proportions are calculated from unique reads only, dereplicated at 100%, not total aligned reads in the data set. The edges of the references were not used in the estimation because edge effects (e.g. skips, residual adapter matches, reference length variation) are reference specific and not generalizable sources of error. However, the fitted relationship applies to the entire reference in the simulated data. [file peerj-09-10539-s002.png]

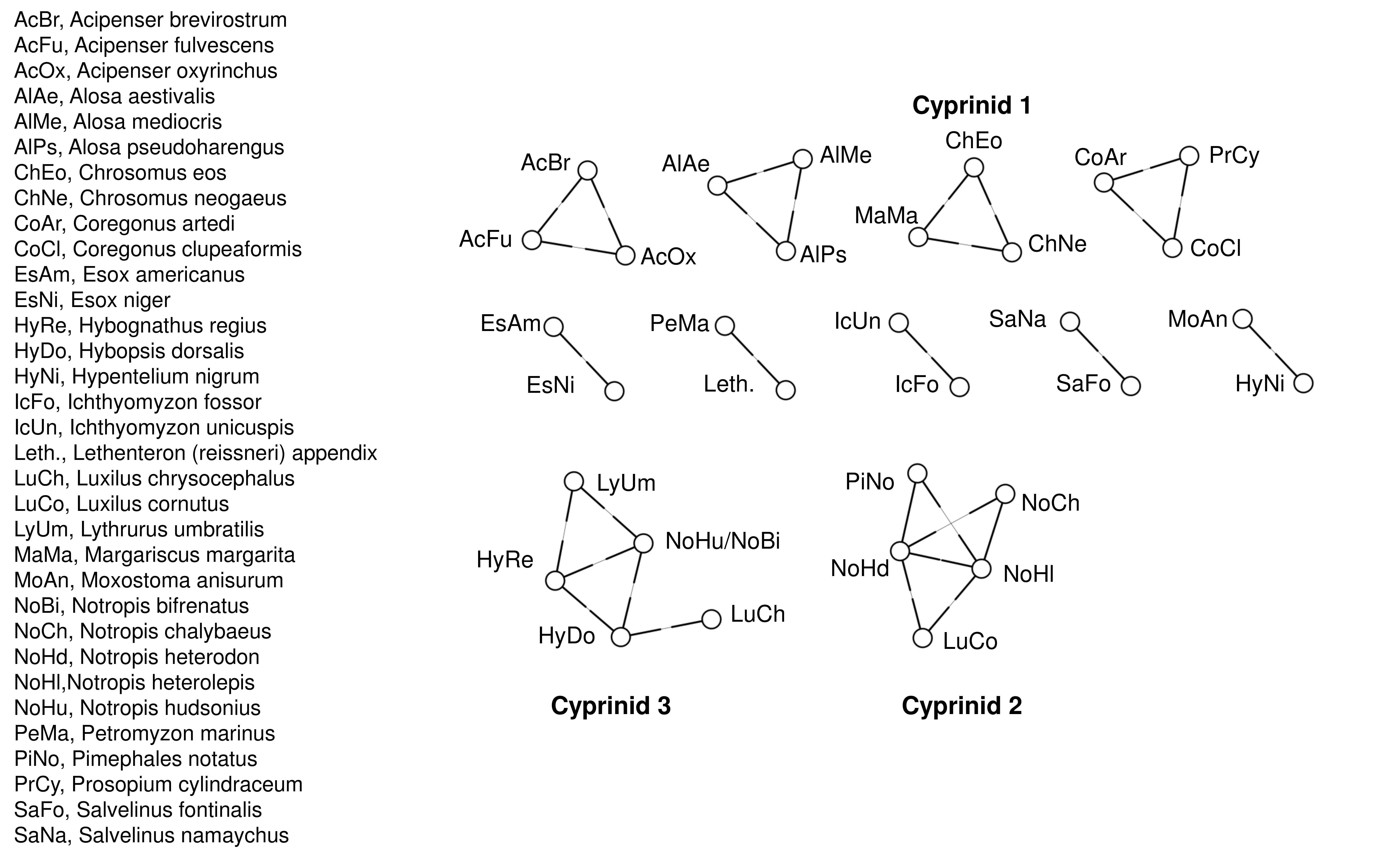

Supplement: Supplemental Information 3 — Networks represent undirected adjacency data (misassignment rates between species pairs). Taxa with error rates greater than 5% were included, along with the taxa that they are conflated with (see Methods for details). [file peerj-09-10539-s003.png]

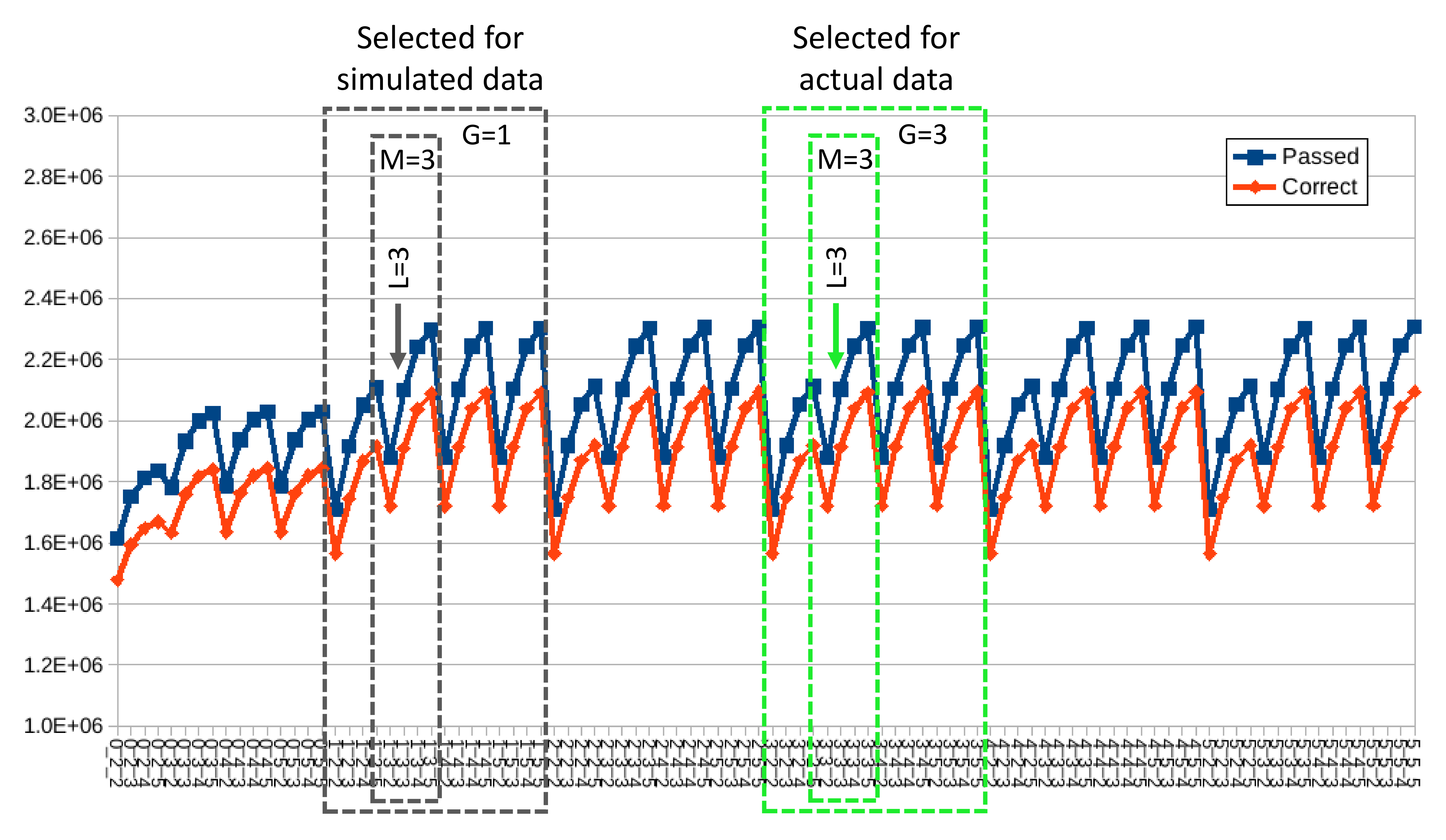

Supplement: Supplemental Information 4 — The horizontal axis lists iterations of the three parameter values investigated, in the form “G_L_M”. For example, “1_3_3” corresponds to G = 1, L = 3, and M = 3. G represents the number of gap positions in the alignment, L is the difference between the reference sequence length and the aligned sequence length, and M is the number of mismatched positions. See text for details. The vertical axis indicates the number of simulated reads mapped. [file peerj-09-10539-s004.png]

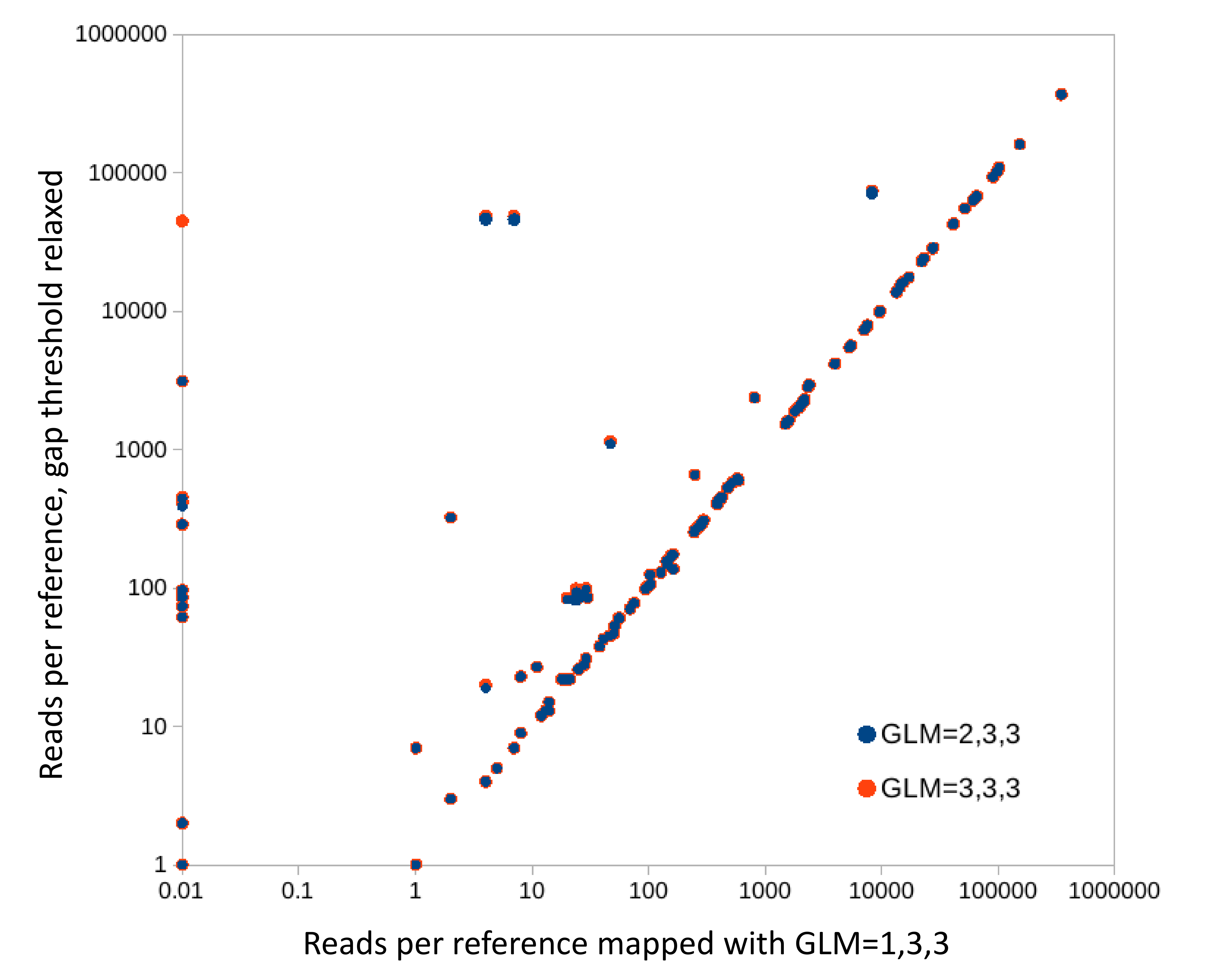

Supplement: Supplemental Information 5 — Axes are log-scaled and indicate the number of simulated reads mapped to source taxa under the thresholds indicated. [file peerj-09-10539-s005.png]

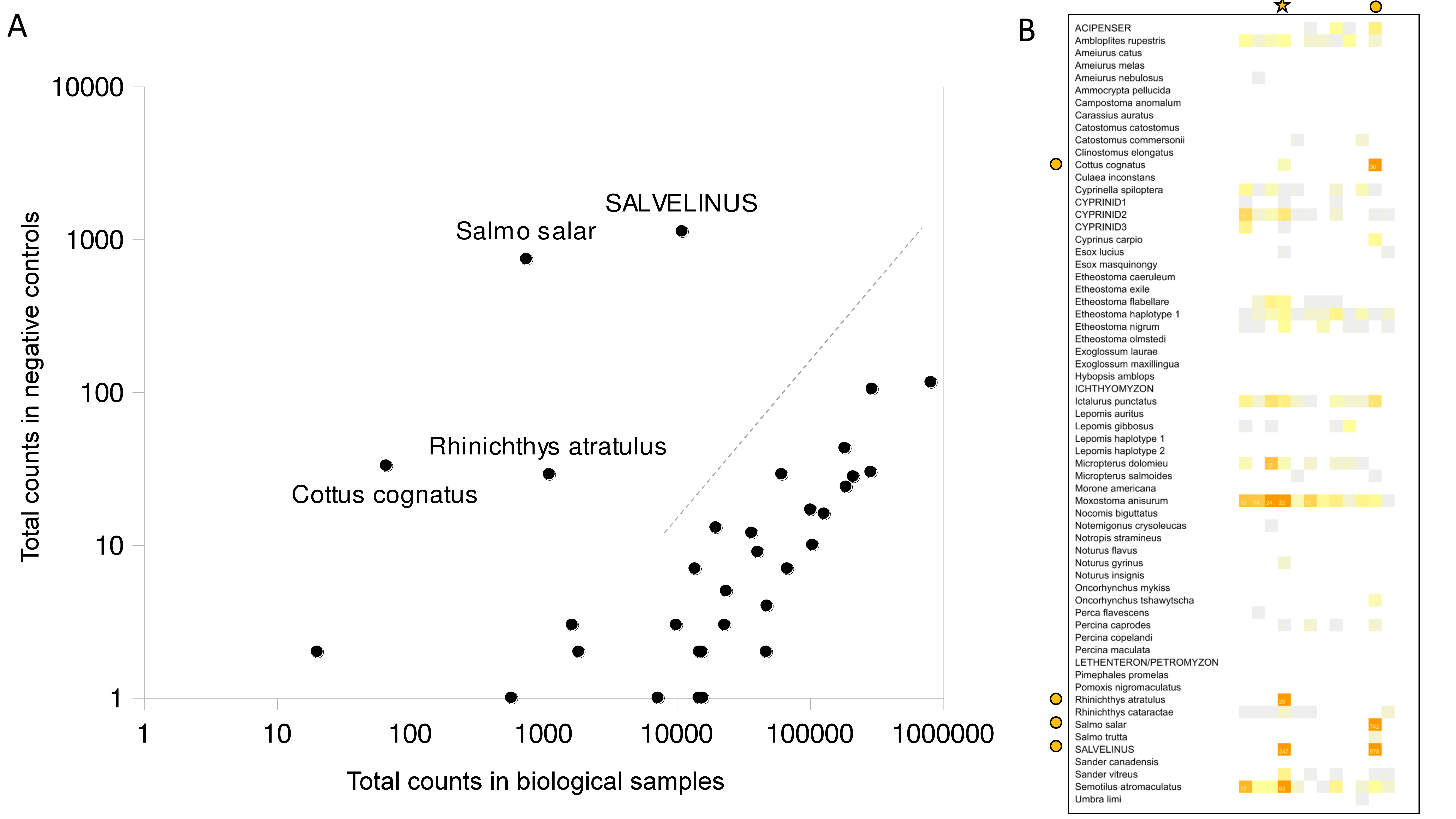

Supplement: Supplemental Information 6 — (A) Scatterplot of total counts for each taxon in biological samples versus negative controls. Dashed line indicates a rate of 1 in 1,000 counts in the combined negative controls relative to the combined biological samples, for illustration purposes only. (B) A heatmap of counts of each taxon in each negative control sample, with darker coloring indicating greater abundance. Taxa and samples presumed to be contaminated are marked by dots. The starred negative-control sample was reported to be potentially contaminated by the field crew. [file peerj-09-10539-s006.png]

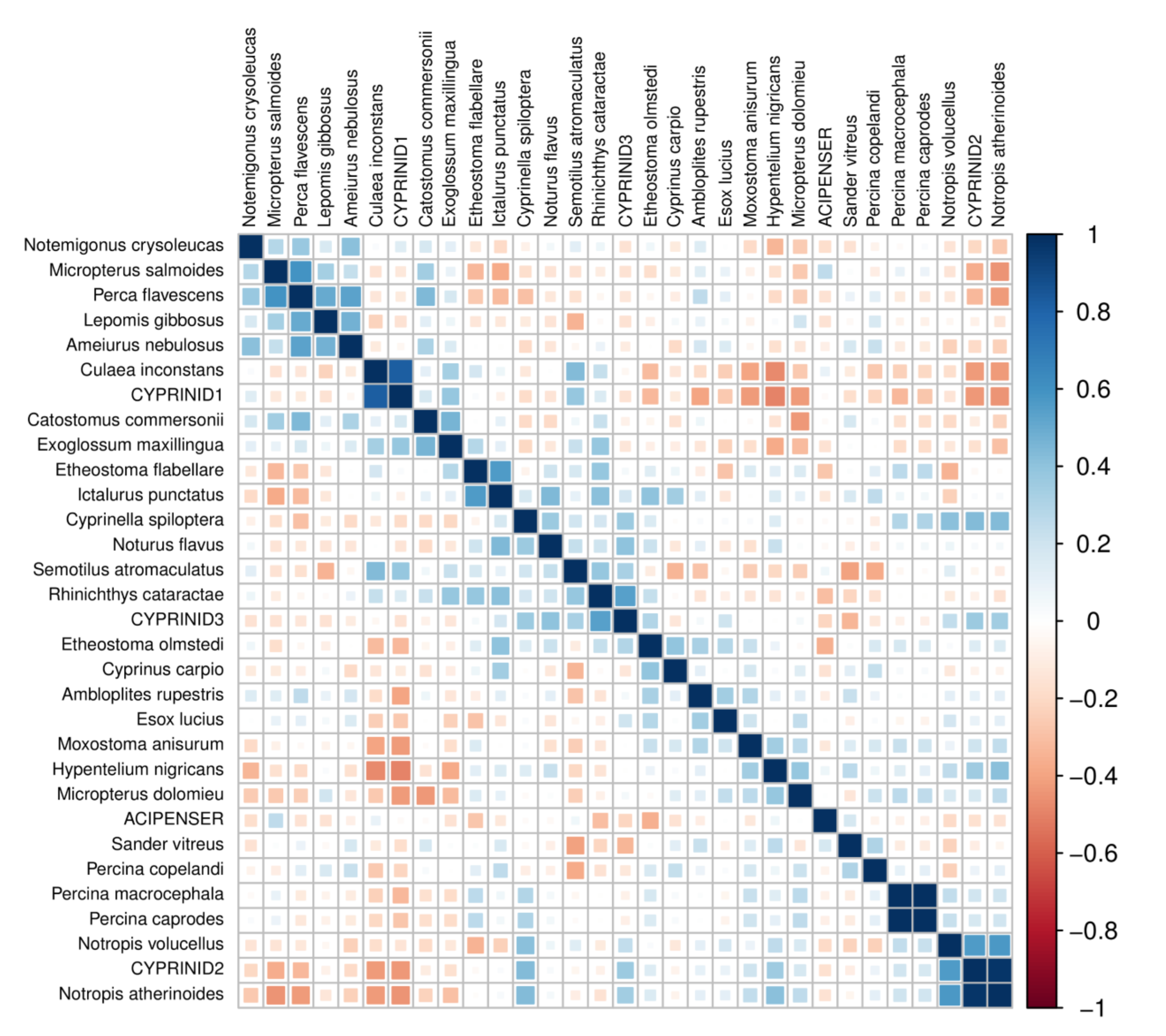

Supplement: Supplemental Information 7 — Only taxa present in at least four samples at a minimum composition of 0.1% were included in this comparison. Spearman’s nonparametric correlation coefficient (r) was specified and the order of samples clustered by Ward’s method with corrplot (Wei and Simko 2017). Yellow boxes highlight pairs of taxa with high correlations coefficients that are discussed in the text. [file peerj-09-10539-s007.png]

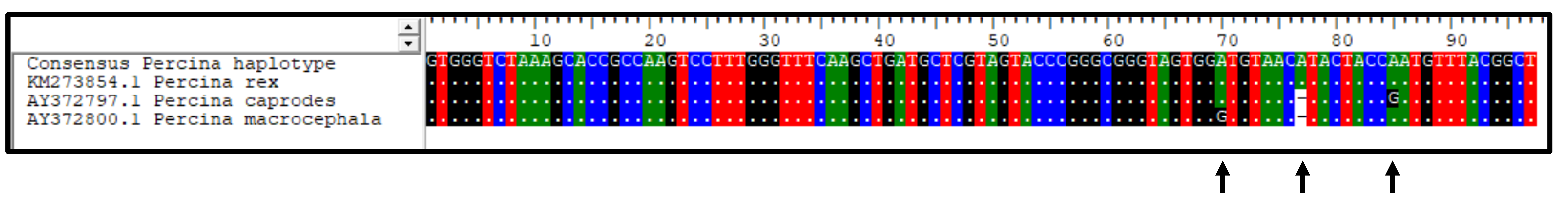

Supplement: Supplemental Information 8 — The first sequence is a representative environmental sequence drawn from the pool mapping to P. caprodes and P. macrocephala. Dots indicate bases that do not vary from the corresponding position in the first sequence. Variant sites are marked by arrows. The environmental sequence is identical to P. rex, which is an endemic of Virgina and not present in New York. The environmental sequence is an edit distance of two from both P. caprodes and P. macrocephala. [file peerj-09-10539-s008.png]

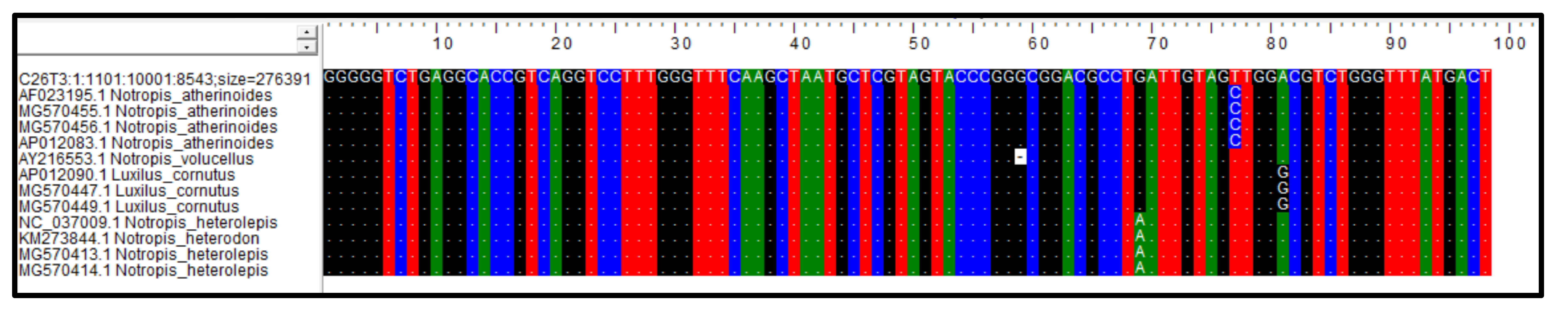

Supplement: Supplemental Information 9 — Dots indicate bases that do not vary from the corresponding position in the first sequence. Reference sequences are invariant within species. The environmental sequence was the cluster representative reported by vsearch after clustering the pool of reads mapping to taxonomic bins with correlated counts (see text for details). [file peerj-09-10539-s009.png]

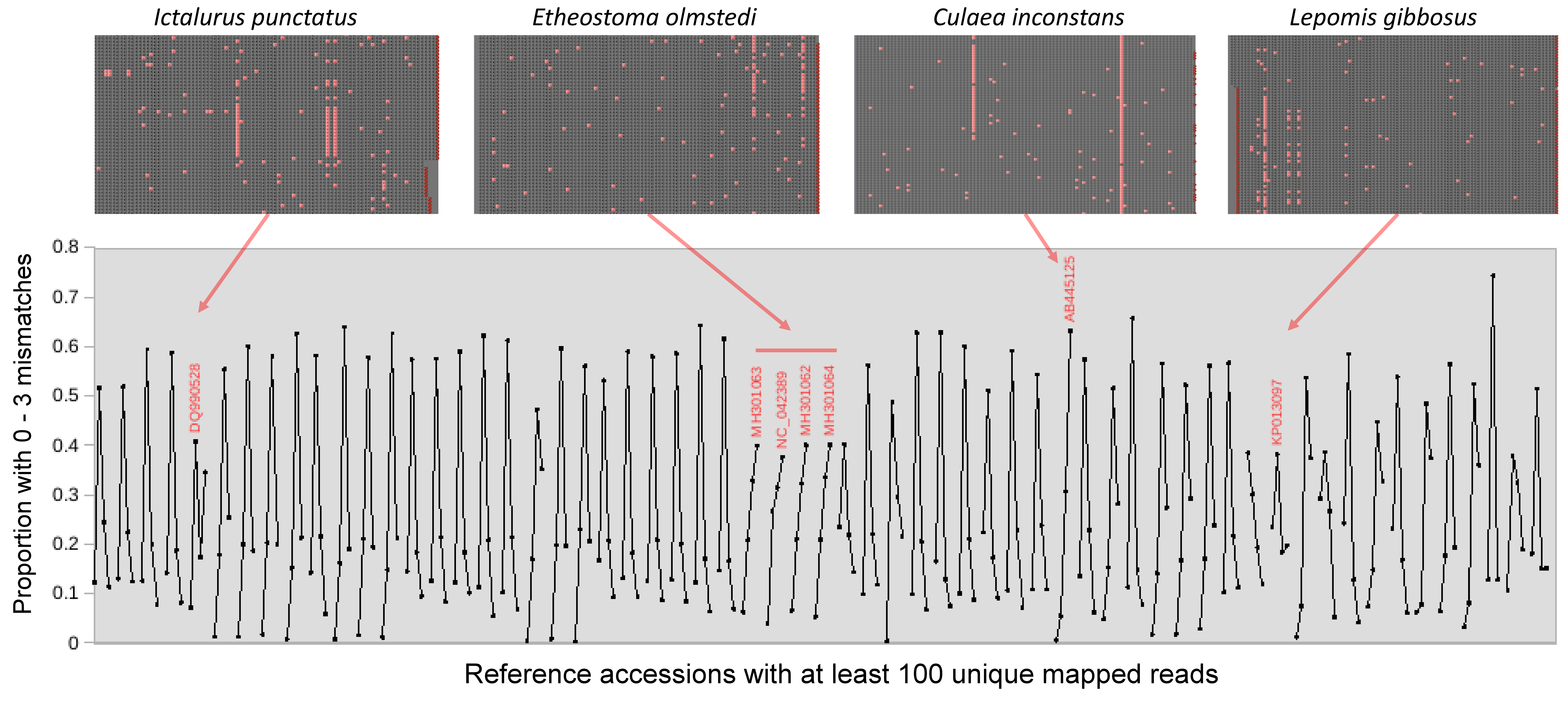

Supplement: Supplemental Information 10 — Mismatch distributions are shown for references with at least 100 mapped reads. Each set of four points represents the proportion of mapped reads with 0 to 3 mismatches, respectively. Most accessions have a single peak, whereas multimodal curves suggest unique haplotypes aligning to the same reference (labelled by accession number and reference taxon). Upper panels illustrate read ‘pileup’ relative to the reference accession, with variant sites colored pink. [file peerj-09-10539-s010.png]

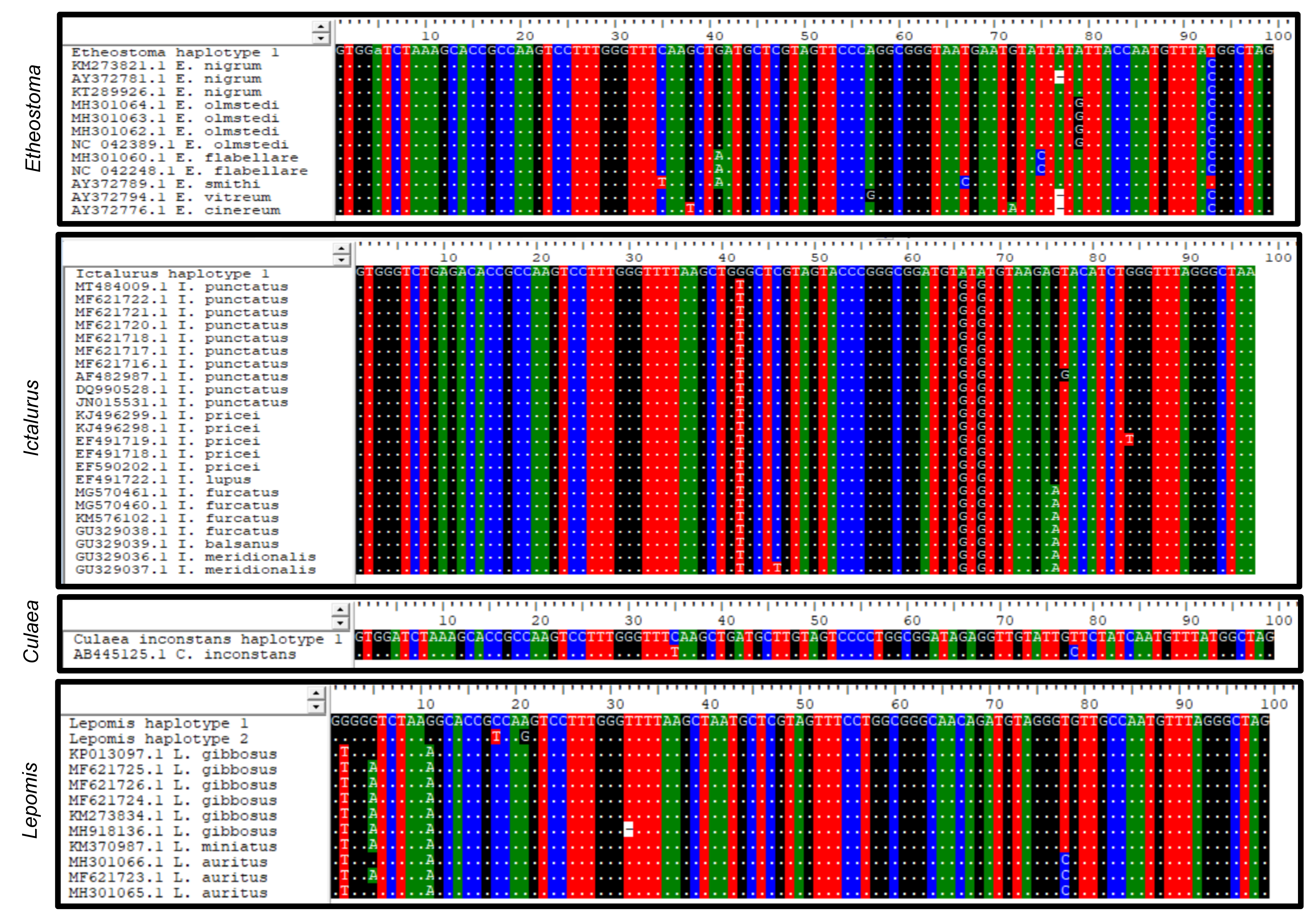

Supplement: Supplemental Information 11 — Taxa correspond to accessions from Figure S11 as well as additional relevant species obtained by BLASTN alignment to NCBI’s nucleotide database. Dots represent bases that do not differ from the first listed sequence in each alignment. For each alignment, haplotypes that are distinct from existing references are indicated. [file peerj-09-10539-s011.png]

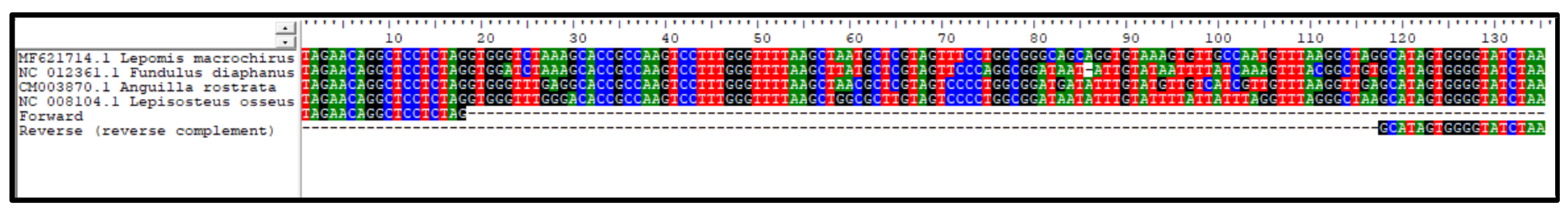

Supplement: Supplemental Information 12 — No mismatches occur in the primer regions (forward and reverse complement primer sequences are indicated in the bottom two rows). [file peerj-09-10539-s012.png]

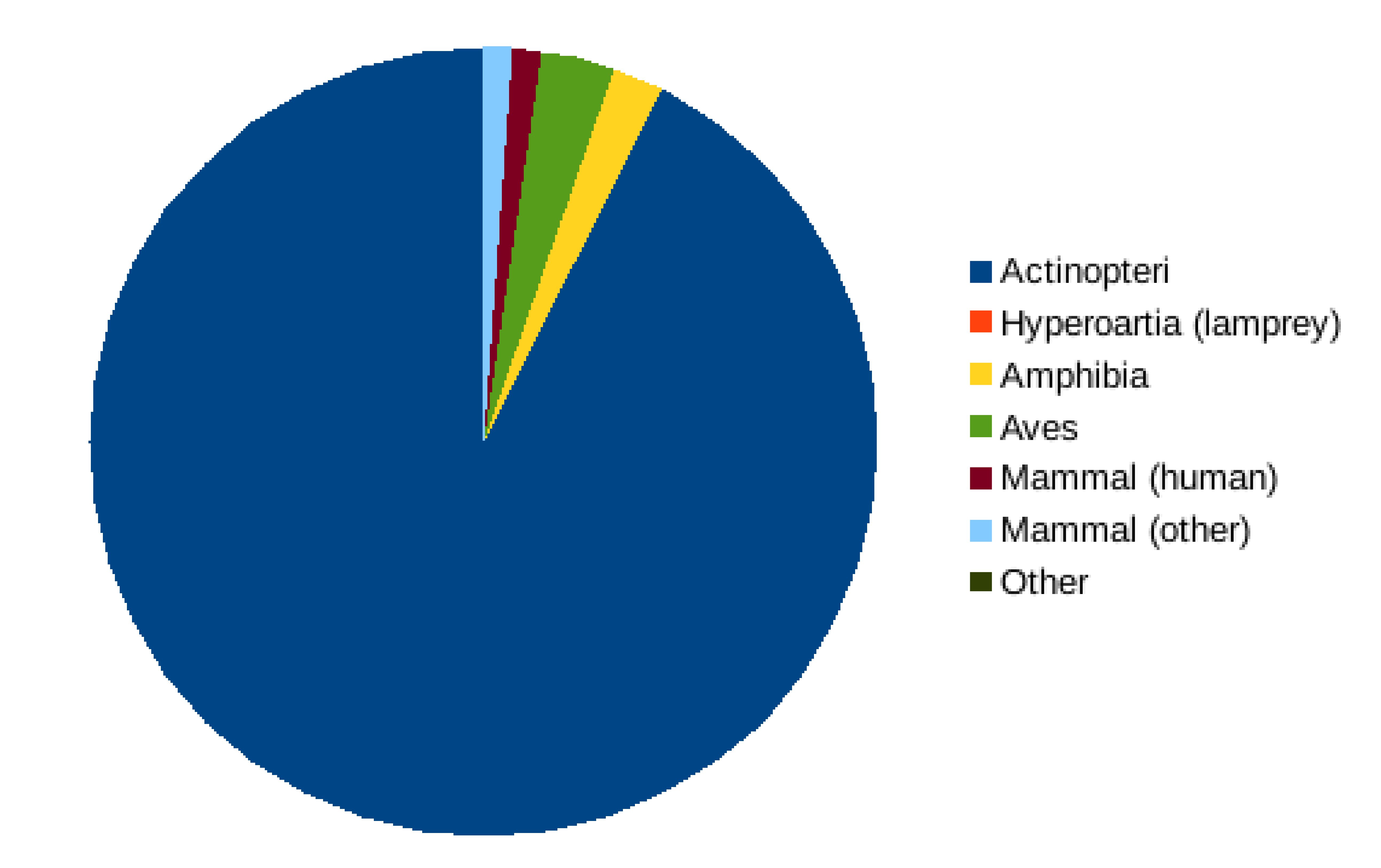

Supplement: Supplemental Information 13 — The majority of unmapped reads are from ray-finned fishes and are presumed to be low-quality reads deriving from the same species as mapped reads. Taxonomic assignments inferred by the lowest common ancestor (LCA) method as described in the Methods. See File S5 for detailed LCA results. [file peerj-09-10539-s013.png]

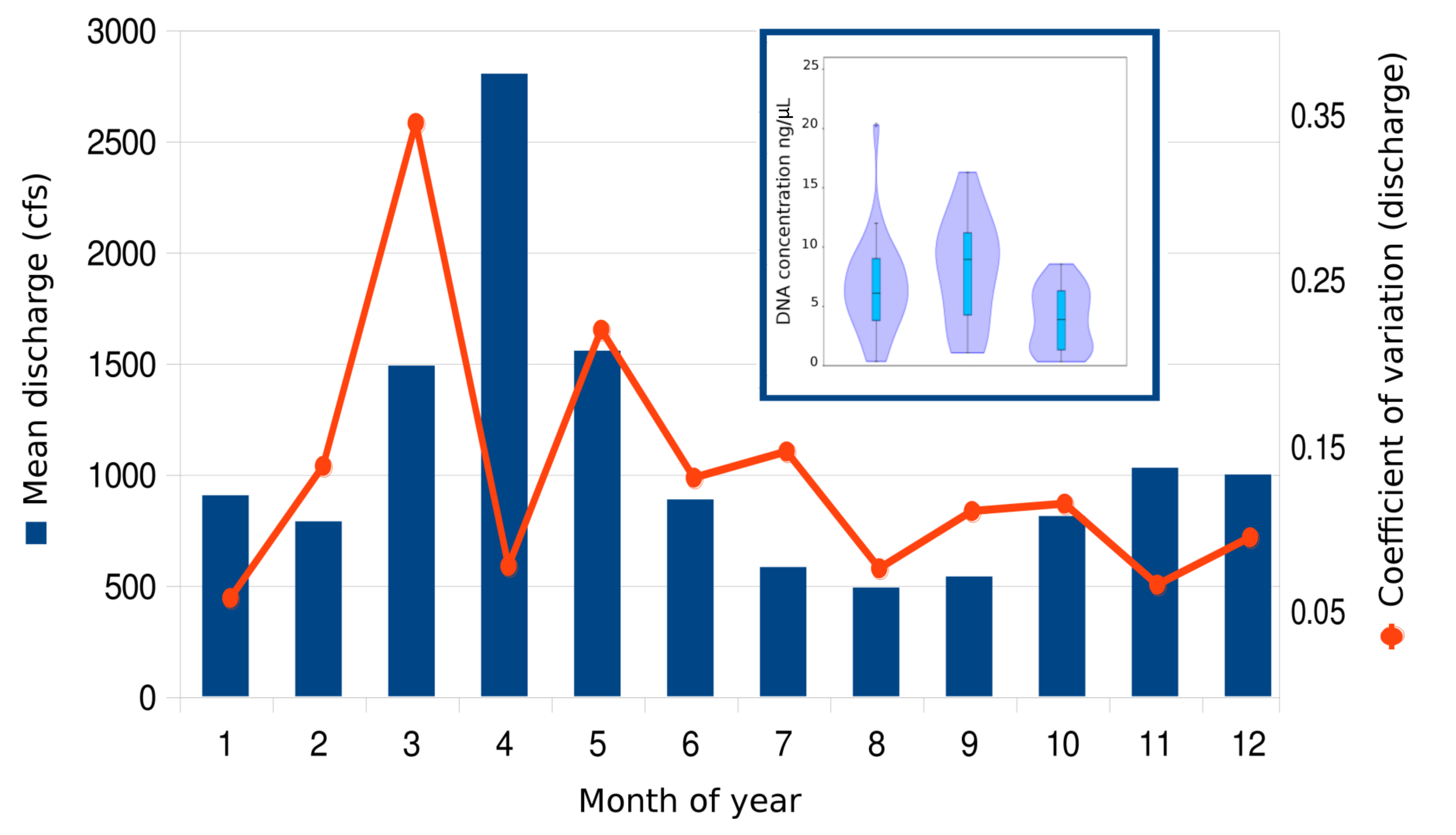

Supplement: Supplemental Information 14 — The primary axis indicates the discharge in cubic feet per second (cfs) since 1917, averaged by month. The secondary axis represents the dispersion around those means, measured as the coefficient of variation (CV). Values are based on an online database of historical records for U.S. Geological Survey water gauge 105838 at Brasher Center, New York. eDNA concentrations obtained by this study are illustrated by violin plot in the inset, by month. Boxes within each violin plot denote quartile values. The outlier eDNA concentration in August was the highest value obtained (20.3 ng/μL) but had a relatively low library yield in both technical replicates (see Figure 3 and text). [file peerj-09-10539-s014.png]

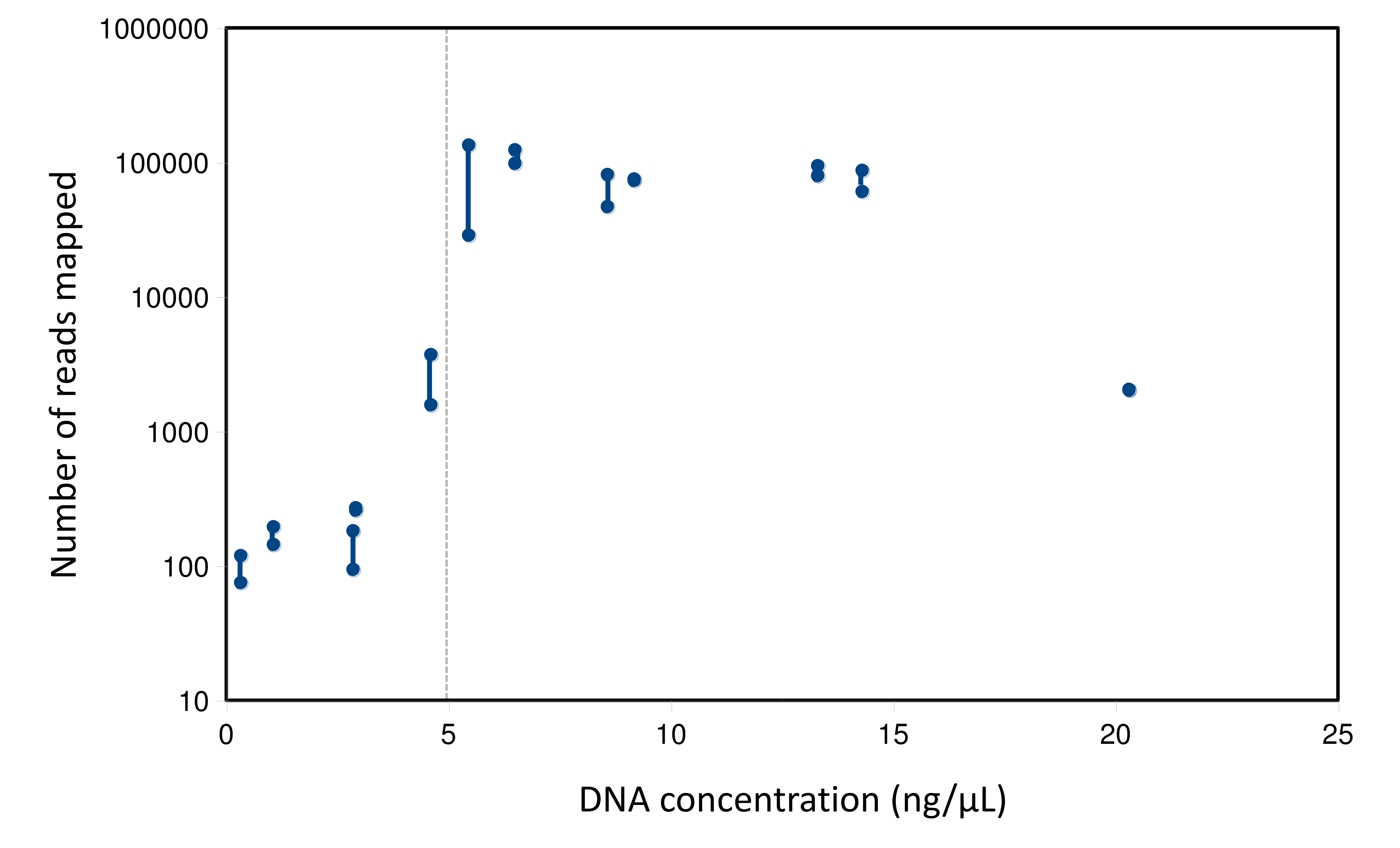

Supplement: Supplemental Information 15 — Twelve technical replicate pairs were performed, represented as dots connected by vertical lines. Each technical replicate had the same source DNA concentration. The vertical axis represents total number of mapped reads (unmapped reads do not contribute to library size). The two replicate pairs with the largest variance are closest to the 5 ng/uL concentration marked on the figure. [file peerj-09-10539-s015.png]

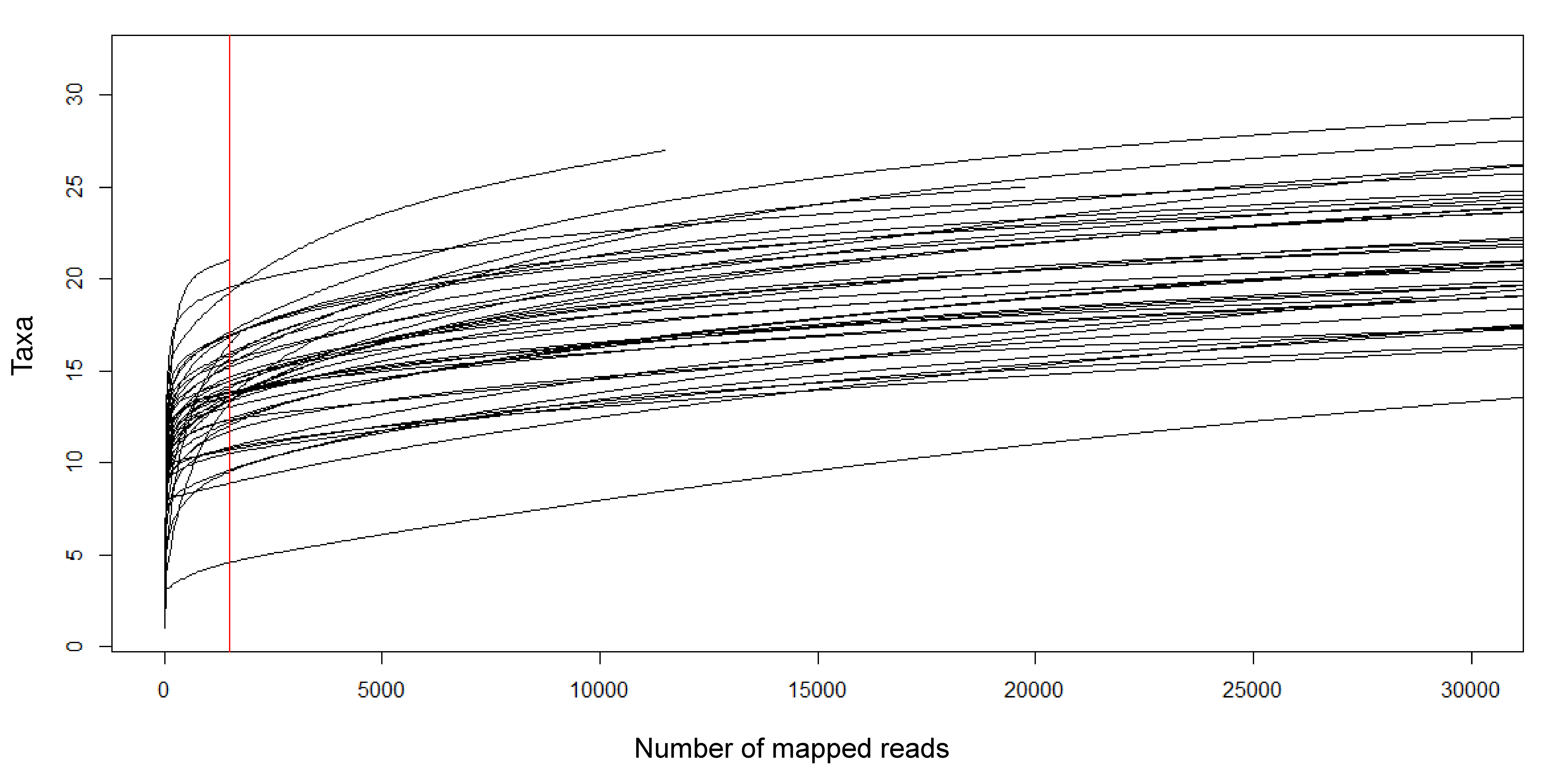

Supplement: Supplemental Information 16 — Red line denotes 1,500 read counts. Taxa are the final species and multispecies bins grouped as described in the text. [file peerj-09-10539-s016.png]

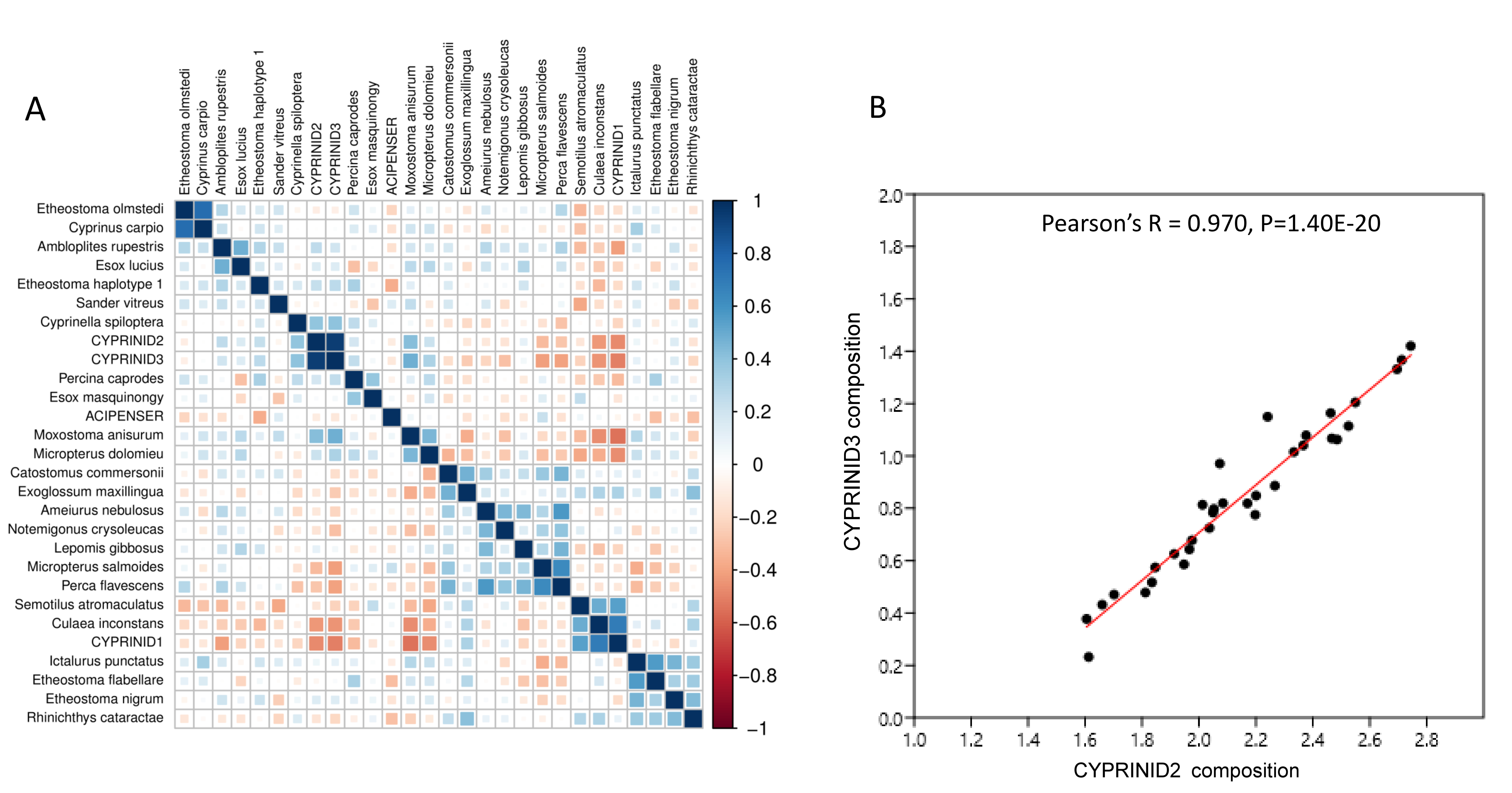

Supplement: Supplemental Information 17 — (A) Correlation matrix of all species and multispecies bins. (B) Correlation between CYPRINID2 and CYPRINID3 compositions for samples in which both were detected. [file peerj-09-10539-s017.png]

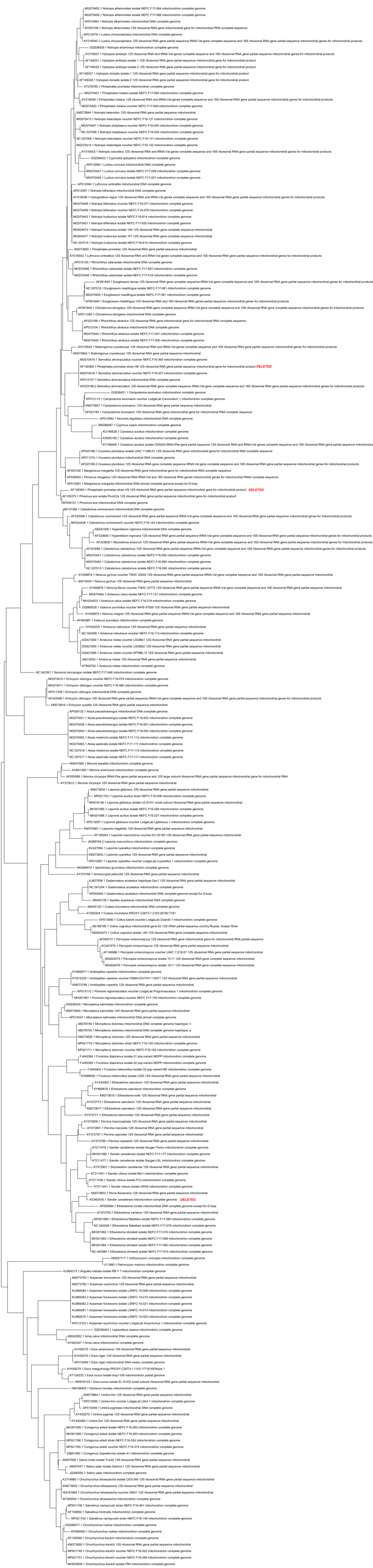

Supplement: Supplemental Information 19 — A neighbor-joining tree used to identify phylogenetically discordant accessions for exclusion from the reference database. Accessions marked “deleted” were considered to be potentially misidentified during submission to NCBI and removed from the reference database. [file peerj-09-10539-s019.pdf]
